# Age of First Exposure to Contact and Collision Sports and Later in Life Brain Health: A Narrative Review

**DOI:** 10.3389/fneur.2021.727089

**Published:** 2021-09-29

**Authors:** Grant L. Iverson, Fionn Büttner, Jaclyn B. Caccese

**Affiliations:** ^1^Department of Physical Medicine and Rehabilitation, Harvard Medical School, Boston, MA, United States; ^2^Spaulding Research Institute, Spaulding Rehabilitation Hospital, Charlestown, MA, United States; ^3^Sports Concussion Program, MassGeneral Hospital for Children, Boston, MA, United States; ^4^Home Base, A Red Sox Foundation and Massachusetts General Hospital Program, Charlestown, MA, United States; ^5^School of Public Health, Physiotherapy and Sports Science, University College Dublin, Dublin, Ireland; ^6^School of Health and Rehabilitation Sciences, The Ohio State University College of Medicine, Columbus, OH, United States; ^7^Chronic Brain Injury Program, The Ohio State University, Columbus, OH, United States

**Keywords:** football, chronic traumatic encephalopathy (CTE), concussion, mild traumatic brain injury (mTBI), repetitive head impacts

## Abstract

A controversial theory proposes that playing tackle football before the age of 12 causes later in life brain health problems. This theory arose from a small study of 42 retired National Football League (NFL) players, which reported that those who started playing tackle football at a younger age performed worse on selected neuropsychological tests and a word reading test. The authors concluded that these differences were likely due to greater exposure to repetitive neurotrauma during a developmentally sensitive maturational period in their lives. Several subsequent studies of current high school and collegiate contact/collision sports athletes, and former high school, collegiate, and professional tackle football players have not replicated these findings. This narrative review aims to (i) discuss the fundamental concepts, issues, and controversies surrounding existing research on age of first exposure (AFE) to contact/collision sport, and (ii) provide a balanced interpretation, including risk of bias assessment findings, of this body of evidence. Among 21 studies, 11 studies examined former athletes, 8 studies examined current athletes, and 2 studies examined both former and current athletes. Although the literature on whether younger AFE to tackle football is associated with later in life cognitive, neurobehavioral, or mental health problems in former NFL players is mixed, the largest study of retired NFL players (*N* = 3,506) suggested there was not a significant association between earlier AFE to organized tackle football and worse subjectively experienced cognitive functioning, depression, or anxiety. Furthermore, no published studies of current athletes show a significant association between playing tackle football (or other contact/collision sports) before the age of 12 and cognitive, neurobehavioral, or mental health problems. It is important to note that all studies were judged to be at high overall risk of bias, indicating that more methodologically rigorous research is needed to understand whether there is an association between AFE to contact/collision sports and later in life brain health. The accumulated research to date suggests that earlier AFE to contact/collision sports is not associated with worse cognitive functioning or mental health in (i) current high school athletes, (ii) current collegiate athletes, or (iii) middle-aged men who played high school football. The literature on former NFL players is mixed and does not, at present, clearly support the theory that exposure to tackle football before age 12 is associated with later in life cognitive impairment or mental health problems.

## Introduction

Playing American football carries inherent risk of sustaining orthopedic injury and concussion (1–4). The rate of sport-related concussion in American tackle football is higher than in most other sports (5–7). Rule changes (8–10), reductions in the number of full contact practices (11–16), protective equipment improvements (17–19), and changes to tackling technique (20–22) have been pursued with the goal of reducing the incidence of sport-related concussion in tackle football (23, 24). Helmet sensor research has revealed that high school and collegiate players are exposed to thousands of head impacts while participating in this sport (25), and concerns have been expressed that repetitive blows to the head might cause long-term changes in brain health (26, 27). Participation in youth tackle football has declined ~10% over the last decade (28), which might reflect, at least in part, concerns among parents about concussion in tackle football and its association with long-term brain health problems.

There are, of course, considerable benefits to participation in youth, high school, and collegiate sports. Participation in sport is associated with diverse health benefits, including better cardiovascular fitness (29), greater lean muscle mass (30), lower rates of obesity (31), lower rates of depression (32, 33) and suicide (34–36), less anxiety and other psychological health problems (37), greater social connectedness (30, 38, 39), and greater self-confidence (39) and self-esteem (40, 41). Notably, greater involvement in sports and exercise is also associated with presumed positive differences in brain neurobiology (42) and better cognitive functioning (42–44) in some studies.

### High School Football and Later in Life Brain Health

Eight studies have examined whether participating in high school football is associated with later in life mental health problems or cognitive impairment. Three research groups have used the National Longitudinal Study of Adolescent to Adult Health database to examine whether boys who played high school football are more likely to have mental health problems during their 20s (45–47). They reported that playing football in high school was not associated with greater lifetime rates of depression (45–47) or anxiety (46), suicidal ideation within the past year (45–47), current symptoms of depression [i.e., within the past seven days (46)], or substance abuse (i.e., nicotine, cannabis, alcohol) (46).

One study surveyed more than 400 middle-aged men (ages 35–55) from the United States general population and reported that those who played high school football were not more likely to have a lifetime history of treatment for mental health problems (48). Middle-aged men who played high school football also reported similar experiences with depression, anxiety, anger, concentration problems, or memory problems in the preceding year compared to men who did not play high school football (48). Four studies with older adult men have also shown no association between playing high school football and later in life problems with brain health. Two research teams examining data from the Wisconsin Longitudinal Study reported no association between playing high school football and later in life cognitive functioning, mental health, or self-rated physical health in older adult men (49, 50). Two medical-record linkage studies found that former high school football players are not at greater risk for later in life neurological or neurodegenerative diseases (51, 52).

### Professional American Football and Later in Life Brain Diseases

Many studies have been conducted with former National Football League (NFL) players. Researchers using diverse experimental neuroimaging techniques have reported that some former NFL players have measurable macrostructural (53–55) and microstructural (56, 57) differences in their brains, and differences in neurochemistry, measured by magnetic resonance spectroscopy (58), and neurophysiology, measured using several technologies (e.g., positron emission tomography and functional magnetic resonance imaging) (59–64). Some clinical studies have reported that some former NFL players perform worse on neuropsychological tests than control participants (54, 57, 65). In large survey studies, most participants report that they have broadly normal health, but a subgroup of former NFL players reports poor mental health and cognitive functioning (66–71). Post-mortem brain donation studies have revealed diverse microscopic neuropathology in some former NFL players (72), including chronic traumatic encephalopathy neuropathologic changes (73, 74).

Studies based on reviews of death certificates have reported greater rates of Alzheimer's disease (75) and amyotrophic lateral sclerosis (75) as contributory causes of death in former NFL players compared to men from the general population, but not psychiatric illness (76) or suicide (76, 77). However, two studies examining death certificates from former NFL players who played between 1959 and 1988 (76) and between 1986 and 2012 (78), found no significant increased risk for “diseases of the nervous system or sense organs.”

A recent mortality study compared former NFL players to former professional Major League Baseball (MLB) players and revealed a greater risk for all-cause neurodegenerative diseases in the former football players (Hazard Ratio, HR = 2.99; 95% CI, 1.64–5.45) (79), although the absolute rates of having a neurodegenerative disease listed on their death certificates were relatively low (i.e., 7.5 vs. 3%). The NFL players, compared to MLB players, had a significantly elevated mortality rate from Parkinson's disease (14/517, 2.7% compared to 5/431, 1.2%). Mortality rates from dementia and/or Alzheimer's disease (16/517; 3.1%) and amyotrophic lateral sclerosis (10/517; 1.9%) were higher in former NFL players than in former MLB players, but differences were not statistically significant. Taken together, the above studies suggest partially elevated risk for former NFL players but not former high school football players.

### Theory: Exposure to Football Before Age 12 and Long-Term Brain Health

A theory proposes that playing tackle football before the age of 12, vs. after that age, causes later in life brain health problems. This theory arose from a small study of retired NFL players conducted at Boston University as part of a program of research entitled “DETECT” (Diagnosing and Evaluating Traumatic Encephalopathy Using Clinical Tests), which identified a statistically significant association between worse neuropsychological functioning and starting to play tackle football before the age of 12 (80–83). These studies served as the impetus for several subsequent studies of high school (84–86), collegiate (84, 87–90), and former NFL players (71, 91–94) (Table 1).

**Table 1:** Summary of studies of retired athletes.

**First author**	**Year published**	**Primary study site**	**Total *N***	**Played before age 12**	**Age (years)**	**Sample/outcome measures**	**AFE binary vs. continuous**	**Positive Findings**	**Negative Findings**
Stamm (80)	2015	BU CTE Center	42	21	*M* = 52, SE = 1	Former NFL Players (DETECT)/Neuropsychological Testing	Binary	Yes	Yes
Stamm (81)	2015	BU CTE Center	40	20	*M* = 52, SD = 6	Former NFL Players (DETECT)/Neuroimaging (DTI; Corpus Callosum)	Binary	Yes	Yes
Schultz (82)	2018	BU CTE Center	86	Not reported	*M* = 55, SD = 8	Former NFL Players (DETECT)/Neuroimaging (Thalamic Volume)	Continuous	Yes	Yes
Kaufmann (83)	2021	BU CTE Center	63	Not reported	*M* = 56, SD = 8	Former NFL Players (DETECT)/Neuroimaging (Cortical Thickness)	Continuous	Yes	Yes
Alosco (92)	2017	BU CTE Center	214	101	*M* = 51, SD = 13	Former Amateur and Professional Football (LEGEND)/Neuropsychological Testing and Self-Report Measures	Binary and Continuous	Yes	Yes
Montenigro (93)	2017	BU CTE Center	93	Not reported	*M* = 47, SD = 14	Former High School and Collegiate Football (LEGEND)/Neuropsychological Testing and Self-Report Measures	Binary	No	Yes
Alosco (94)	2018	BU CTE Center	211	84	*M* = 63, SD = 18	Deceased Former Amateur & Professional Football (UNITE)/Post-Mortem Neuropathology, Symptoms, Age of Onset of Problems	Binary and Continuous	Yes	Yes
Solomon (91)	2016	Vanderbilt University	45	Not reported	*M* = 47, SD = 9	Former NFL Players/Neuroimaging, Neuropsychological Testing, Self-Report Measures	Continuous	No	Yes
Roberts (71)	2019	Harvard University	3,506	Not reported	*M* = 53, SD = 14	Former NFL Players (Football Players Health Study)/Self-Report Measures	Binary and Continuous	No	Yes
Iverson (86)	2020	Harvard Medical School	123	62	*M* = 45, SD = 6	Former high school football players/Self-Report Measures	Binary and Continuous	No	Yes
Iverson (85)	2021	Harvard Medical School	186	87	*M* = 52, SD = 11	Former high school football players/Self-Report Measures	Binary and Continuous	No	Yes
Bryant (122)	2020	Cleveland Clinic Lou Ruvo Center for Brain Health	Active fighters (*n* = 442); Retired fighters (*n* = 64)	Not reported	Active fighters *M* = 29 SD = 5; Retired fighters; *M* = 48, SD = 10	Male and female licensed professional fighters (boxers, mixed martial artists, and martial artists); Professional Fighters Brain Health Study/Neuroimaging, Neuropsychological Testing, Balance, Self-Report Measures	Continuous	Yes	Yes
Hunzinger (121)	2021	University of Delaware	1,034 (Active and Retired)	753	*M* = 32, SD = 11	Current and former male and female rugby players/Self-Report Measures	Binary and Continuous	No	Yes

*Full summaries of these articles are in the online supplement. BU, Boston University; CTE, Chronic traumatic encephalopathy; NFL, National Football League; DETECT, Diagnosing and Evaluating Traumatic Encephalopathy Using Clinical Tests; LEGEND, Longitudinal Examination to Gather Evidence of Neurodegenerative Disease; UNITE, Understanding Neurologic Injury and Traumatic Encephalopathy; AFE, age of first exposure; DTI, diffusion tensor imaging*.

Since 2018, several states, such as New York, Illinois, California, Maryland, New Jersey, and Massachusetts have introduced legislation to ban tackle football (i) entirely from youth sports or (ii) prior to a certain age (95). Advocacy groups for this legislation have cited this early study to support the campaign that playing tackle football before age 12 is associated with brain health problems later in life (80, 92, 94).

There is a need to critically review the literature related to this theory because existing studies have notable methodological limitations. Furthermore, the results of the original, theory-generating study have not been replicated by other research groups. Research on age of first exposure (AFE) to contact/collision sport and later in life brain health has seen significant progress in recent years, in study design, methodology, and subsequent study findings. The purpose of this narrative review is to (i) discuss the fundamental concepts, issues, and controversies surrounding existing research on AFE to contact/collision sport, and (ii) provide a balanced interpretation, including risk of bias assessment findings, of this body of evidence. We used PubMed for our initial search and a pearl growing strategy to find additional articles, and then used the Quality in Prognostic Studies (QUIPS) assessment tool to evaluate the risk of bias of included studies. This review is divided into four sections, as follows: Origin of the Theory, Studies with Retired Amateur and Professional Athletes, Studies with Current Athletes, and Risk of Bias Assessment Findings.

## Methodology

We performed article searches in PubMed using both (i) keywords that are relevant to the narrative review question under investigation (e.g., “age of first exposure,” “AFE,” “sport,” “football,” “repetitive head impact”), and (ii) the names of authors who have published scientific articles on this topic. Additionally, we used a pearl growing strategy, similar to snowball sampling, whereby the reference lists of each study were reviewed to identify other studies that were relevant to this review. For each study identified, we used the ‘find similar articles' function in PubMed to retrieve related scientific articles answering conceptually similar research questions. We searched only for articles published from 2015 as the original scientific article proposing the theory of AFE (and its relationship with later in life brain health) stems from an article published in 2015 (80).

Twenty-one original research studies investigating the relationship between AFE to contact and/or collision sports and later in life brain health were identified and included for narrative review. Narrative reviews, presented in a systematic manner, are useful as a scholarly summary, interpretation, and critique of studies that answer a common research question while using distinctive methodologies (96). These reviews are also helpful for providing a historical account of the development of a theory or research on a topic (97). Two authors (FCB and JBC) independently extracted data from included studies using a pre-developed data extraction template that focused on study characteristics, including empirical design, participants, exposure definition(s), outcome type(s) and definition(s), and potential confounders. The extraction table that summarizes all of the articles is included as an Online Supplementary Material.

We used the QUIPS assessment tool to evaluate the risk of bias of studies included in this narrative review. QUIPS is an outcome-level, domain-based tool developed by Hayden et al. to evaluate the risk of bias in prognostic factor studies (98). The QUIPS has six risk of bias domains that outline potential sources of bias in a prognostic factor study: (i) study participation, (ii) study attrition, (iii) prognostic factor measurement, (iv) outcome measurement, (v) confounding, and (vi) statistical analysis and reporting. Due to the cross-sectional nature of included studies and lack of participant follow-up, we omitted study attrition from our risk of bias assessment. Risk of bias domains are judged to be at *low, moderate*, or *high* risk of bias, which in turn inform an overall judgement of *low, moderate*, or *high* risk of bias for each study. For example, if all risk of bias domains in a study were at *low* risk of bias, an overall judgment of *low* risk of bias was considered. If there was at least one domain at *moderate* risk of bias, an overall judgment of *moderate* risk of bias was considered. If there was at least one domain at *high* risk of bias or multiple domains at *moderate* risk of bias, an overall judgment of *high* risk of bias was considered.

Using QUIPS prior to data extraction, two review authors (FCB & JBC) developed content-specific criteria within each risk of bias domain relating to the relationship between AFE and later-life cognitive, behavioral, and mental health outcomes (see the QUIPS tool in the Supplementary Material). All review authors approved AFE-specific risk of bias criteria. Two assessors (JBC & FCB) independently performed domain-based risk of bias assessments of each study. Between-assessor disagreement was resolved via discussion. A third author (GLI) arbitrated persisting between-assessor disagreement that could not be resolved via discussion.

## Origin of the Theory

Stamm et al. (80) studied a sample of 42 retired NFL players, half of whom began playing football before the age of 12 (*n* = 21) and half of whom began after the age of 12 (*n* = 21). Both groups were selected from a larger sample of retired NFL players (*N* = 74), all of whom self-reported cognitive, behavioral, and mood symptoms for at least 6 months prior to enrolment in the study. For the DETECT study, a battery of neuropsychological tests measuring attention (e.g., Digit Span), speed of processing (e.g., Trails A and Digit Symbol Coding), visual-spatial and visual-constructional skills (e.g., Rey Complex Figure and Map Reading), confrontation naming, learning and memory (e.g., List Learning and Story Learning), verbal fluency (e.g., Controlled Oral Word Association Test and Animal Naming), and executive functioning (e.g., Trails B, Wisconsin Card Sorting Test, and the Color-Word Interference Test) was administered to each of the retired NFL players. The authors reported that they selected a “focused set” of neuropsychological outcome measures for the study to reduce the likelihood of type I errors and based on their *a priori* hypotheses. They selected only two neuropsychological tests from the battery, the Wisconsin Card Sorting Test and the List Learning Test. They also reported the results from a word reading test that they noted is commonly used in research to estimate premorbid (i.e., longstanding) verbal intellectual ability. The authors hypothesized “that those who began playing football before age 12 would perform significantly worse on measures of executive function, memory, and premorbid estimated verbal IQ (eVIQ) than those who started playing at age 12 or older.” They reported that those who started playing at a younger age performed worse on both of their selected neuropsychological tests and the reading test. The authors concluded that these differences were likely due to greater exposure to repetitive neurotrauma during a developmentally sensitive maturational period in their lives (80).

### Methodological Issues Relating to the Original Study

Other researchers have expressed important concerns about methodological problems and the conclusions drawn from this study (99–101), and some of these concerns were reported in the discussion section of the original article and by the authors in a letter to the editor (102). First, the study was small and potentially under-powered, containing only 21 subjects in each group. Second, the sample is not representative of the general youth football player because most youth players do not go on to play professional football and because participants self-reported an astonishing average of nearly 400 prior concussions. Third, it is not clear in the study whether the choices of using the two neuropsychological tests from all the other tests, or a binary cutoff threshold of age 12, were influenced by exploratory analyses and selective outcome reporting. Increased risk for spurious findings occurs when researchers undertake exploratory analyses (103, 104) and/or hypothesize after the results are known (105, 106). Finally, the groups differed on the presence of learning disabilities, with the younger AFE group having more subjects with lifelong learning disabilities. It is well-known that people with learning disabilities, as a group, perform worse on cognitive tests (107–110).

In this original study, the younger AFE group performed worse on a word reading test. The authors argued that this was not a coincidental difference between the groups but rather this difference was *caused* by playing football (80). Given the complex nature of determinants of reading ability, inferring causality from reductive statistical approaches using potentially underpowered and unrepresentative samples increases the likelihood that study conclusions are inaccurate. Additionally, the authors did not cite any studies to support this opinion that playing football before age 12 causes learning disabilities or somehow interferes with a person's lifelong proficiency in single word reading, and we are not aware of any studies that support these assertions. In contrast, a recent study suggested that neurotrauma exposure variables explained <1% of the variance on a word reading test, whereas sociodemographic and academic aptitude variables explained >20% of the variance in a sample of 6,598 collegiate student-athletes (111). Thus, a more parsimonious and logical explanation is that differences in rates of learning disabilities, and lower reading scores, were not caused by playing the sport at an earlier age but rather reflect a longstanding, lifelong difference between both (small) samples of former NFL players. This is fundamentally important because it is well-established in neuropsychology that single-word reading performance is positively correlated with both intelligence and performance on tests in other cognitive domains, including memory and executive functioning (112–119). As such, a compelling alternative explanation for the findings from the study by Stamm et al. is that cognitive test scores were worse because the earlier AFE group was more likely to have learning disabilities and/or lower reading skills, not because they played football starting at a younger age. That said, the original authors reported in a letter to the editor that the statistically significant difference between the two groups on the learning test and the executive function test remained after using the reading test as a covariate (102).

## Studies With Retired Amateur and Professional Athletes

Three additional studies by the same research group were published, using mostly the same sample of retired NFL players. These studies revealed (i) differences in white matter microstructure (81), as measured by diffusion tensor imaging (DTI), (ii) differences in thalamic volumes (82) and (iii) differences in cortical thickness (83), as measured by magnetic resonance imaging (MRI). Specifically, Stamm et al. (81) examined fractional anisotropy, trace, axial diffusivity, and radial diffusivity in the whole corpus callosum and in five sub-regions. Former NFL players in the AFE <12 group had significantly lower fractional anisotropy in three corpus callosum sub-regions and higher radial diffusivity in one corpus callosum sub-region than those in the AFE ≥12 group. Thus, of the 24 analyses that the authors performed in this study, 4 (17%) were significant. Schultz et al. (82) reported that right thalamic volume, but not left thalamic volume, was associated with AFE, whereby for every year a participant started playing football earlier, the average decrease in right thalamic volume was 64.9 mm^3^, but thalamic volumes were mostly unrelated to cognitive and behavioral/mood assessments. Finally, Kaufmann et al. (83) identified clusters of cortical thickness that were associated with AFE. Specifically, a statistically significant correlation between AFE and cortical thickness was found in the left supramarginal gyrus and superior parietal lobule, in the right posterior superior frontal cortex and dorsal precentral gyrus, as well as in the bilateral cuneal cortex and pericalcarine cortex and lingual gyrus. Thus, there have been four published studies using the sample of retired NFL players recruited for the DETECT study, and all have found some significant findings in brain structure or microstructure that were associated with earlier AFE. However, the number of brain regions examined, and the number of statistical comparisons undertaken, were not always clear. Moreover, researchers should not assume that these are four *independent* studies given that there was likely considerable overlap in subjects across the studies.

The Boston University Chronic Traumatic Encephalopathy (CTE) Center recruited an independent cohort of tackle football players for the “Longitudinal Examination to Gather Evidence of Neurodegenerative Disease” (LEGEND) study. Participants in the LEGEND study included former football players older than 18 years of age with a history of participation in organized sport, including high school, college, or professional levels of play. Alosco et al. (92) examined the association between AFE to tackle football and later in life cognitive and neuropsychiatric outcomes in former high school (*n* = 43), college (*n* = 103), and professional (*n* = 68) tackle football players from the LEGEND Study. Those with younger AFE, whether analyzed continuously or dichotomously (using the age 12 cutoff), self-reported worse executive function, depression, and apathy, but AFE was unrelated to cognitive functioning as assessed by the Brief Test of Adult Cognition. However, when examining the effects of cumulative head impact exposure among subjects from the same LEGEND cohort, Montenigro et al. (93) reported that AFE did not add independently to the model, nor did including AFE in the model eliminate the significance of cumulative head impact exposure for predicting clinical outcomes, suggesting that cumulative exposure may affect neuropsychiatric and cognitive outcomes more than AFE. The authors noted that participants with AFE <12 had some increase in the risk for impairment, but this was not statistically significant for any outcome measure after adjusting for cumulative exposure. Therefore, in the Montenigro et al. study (93), unlike the Alosco et al. study (92), AFE was not related to self-reported worse executive function, depression, and apathy.

Four follow-up studies, using different samples and methodologies, have not replicated findings from the DETECT and LEGEND studies regarding AFE (71, 85, 86, 91). Solomon et al. (91) aimed to replicate the study findings of Stamm et al. with data from former NFL players (120), focusing particularly on neuropsychological test results. There were 45 retired NFL players who underwent extensive medical history, neurological examination, Mini-Mental State Examination, Beck Depression Inventory, Patient Health Questionnaire, magnetic resonance imaging (MRI), APoE genotyping, and paper and pencil and computerized neuropsychological testing. Unlike participants in the Stamm study (80), retirees reported 9.0 ± 6.9 concussions (maximum = 25) and 14.9 ± 7.9 “dings,” which were likely unrecognized concussions. Exclusion criteria for retirees were also more stringent than in the study by Stamm et al. Notably, individuals with a history of significant alcohol abuse and/or drug abuse were excluded, whereas over 50% of participants in DETECT reported illicit drug and alcohol use. Solomon et al. reported no relationship between years of pre-high school tackle football exposure and any neuroradiological, neurological, behavioral, or neuropsychological outcomes.

Roberts et al. (71) examined survey data from a large cohort of former NFL players (*N* = 3,506) who participated in the NFL after 1960. Participants completed the short form of the Quality of Life in Neurological Disorders: Applied Cognition–General Concerns (Neuro-QOL), to assess perceived cognitive functioning, and the Patient Health Questionnaire−4 (PHQ-4) to assess symptoms of depression and anxiety. Among this very large cohort, there was no relationship between AFE and subjectively experienced cognitive problems, depression, or anxiety, whether AFE to organized tackle football was analyzed continuously or dichotomously at age 12. In contrast, greater seasons of professional play were associated with worse perceived cognitive functioning, depression, and anxiety. Thus, in the largest study of former NFL players to date, earlier AFE to organized tackle football was not related to later in life brain health problems.

Former NFL players are not ideal for examining associations between youth sports participation and later in life brain health. Iverson et al. (85, 86) recruited two independent samples of men in the United States: (i) ages 35–55 years (*M* = 44.8, SD = 6.2 years; *N* = 123) and (ii) ages 35 and older (*M* = 51.8, SD = 10.9 years; *N* = 186), who played high school football. Participants completed the Patient Health Questionnaire-8 to assess symptoms of depression over the preceding 2 weeks, the British Columbia Post-Concussion Symptom Inventory to assess frequency and severity of persistent concussion symptoms, and a survey of medical history. Although men who played football before age 12 reported a greater number of lifetime concussions and a younger age at first concussion than those who started at or after age 12, younger AFE to football was not associated with symptoms of, or treatment for, mental health problems, memory loss, chronic pain, or headaches. Specifically, younger AFE to football was not associated with (1) ever having seen a psychologist, counselor, or therapist for mental health care; (2) ever being prescribed medications for depression; (3) feeling depressed in the preceding week or year; (4) current symptoms of depression; (5) ever having been prescribed medications for anxiety; (6) having had problems with anxiety in the preceding week or year; (7) current symptoms of anxiety; (8) current post-concussion-like symptoms; or (9) current perceived difficulties with cognitive functioning (85, 86).

Hunzinger et al. (121) surveyed 1,034 current and former community-level rugby players. Participants completed patient-reported outcomes, including the Brief Symptom Inventory 18 to assess psychological distress, the Short Form Health Survey 12 to assess physical and mental health quality of life, and the Satisfaction with Life Scale. Earlier AFE to contact/collision sports, whether analyzed continuously or dichotomously at age 12, was not associated with worse psychological distress or quality of life among men or women.

Finally, there has been one large-scale study of active (*n* = 442) and retired (*n* = 64) professional fighters (i.e., boxers, mixed martial artists, and martial artists), and this study examined numerous neuroimaging variables, neuropsychological test scores, and self-report symptom measures. Earlier AFE to combat sports was associated with several differences in brain macrostructure, worse measured cognitive functioning, and greater self-reported symptoms of depression and impulsiveness (122). Although the authors state, “AFE to competitive fighting was defined as the study participant's self-reported age (in years) when competitive fighting began…This age was the earlier of either amateur or professional competitive fighting experience,” the descriptive statistics for AFE were not presented. However, when subtracting the mean years of fighting from the mean age for active fighters, data suggest the AFE for this cohort is 24 years old, so it remains unclear how these findings inform long-term outcomes of repetitive neurotrauma on neurodevelopment. Nonetheless, based on this study, earlier AFE to fighting is associated with smaller brain structures, worse cognitive functioning, and greater subjectively experienced symptoms.

## Studies With Current Athletes

No published studies of current athletes show a statistically significant association between playing football (or other contact sports) before the age of 12 and worse functioning (84, 87, 88, 90, 111, 123, 124) or worse clinical outcome from concussion (89). In contrast, several large-scale cross-sectional studies of high school and collegiate athletes have found that earlier AFE to football and other contact sports is not significantly associated with worse objectively measured cognitive functioning (84, 87, 88, 90, 123) or greater self-reported physical, cognitive, or emotional symptoms (84, 88). Collectively, these five studies examined four independent cohorts amassing nearly 10,000 athletes across 20 different outcome measures, and they did not report a single outcome measure that was worse with earlier AFE to football or other contact sports.

It is important to note that many of these studies included a control group comprising athletes who participated in non-contact sports and that many of these studies controlled for neurodevelopmental history and a variety of sociodemographic factors that may influence both sports participation and outcome measures. For example, several large-scale studies have shown that having a neurodevelopmental problem, such as ADHD (109, 110), learning disability (109, 110), or significant academic problems (107, 108), was associated with lower scores on cognitive testing and greater self-reported physical, cognitive, and emotional symptoms. Lower SES and other sociodemographic disparities were also associated with worse performance on cognitive testing in student athletes (90, 111, 125). These studies are not without limitations, however. Notably, most large cohorts were recruited through a single study, the National Collegiate Athletic Association (NCAA)-US Department of Defense (DoD) Grand Alliance Concussion Assessment, Research and Education (CARE) Consortium Study (87–90, 111, 123), and outcome measures were selected based on those typically included in sport-related concussion baseline assessments, which may not be as sensitive for more subtle effects of early exposure to repetitive head impacts. Furthermore, findings only inform short- to medium-term effects in current athletes who may have sufficient cognitive reserve to mask potential consequences of earlier AFE to repetitive neurotrauma. Thus, if there is an association between earlier AFE and worse mental health or cognitive functioning, that association might emerge in association with aging. Nonetheless, the best available evidence suggests that, in current high school and collegiate athletes, earlier AFE to contact/collision sport is not associated with lower cognitive abilities on objective testing at baseline (84, 87, 88, 90, 123), greater physical, cognitive, or emotional symptom reporting at baseline (84, 88), or worse clinical outcome following concussion (89).

## Risk of Bias Assessment Findings

All studies (*k* = 21, 100%) were judged to be at *high* overall risk of bias (Table 2). In the study participation domain, four studies (19%) were deemed to be at *low* risk of selection bias and two studies (10%) were deemed to be at *moderate* risk of selection bias. Fifteen (71%) were judged to be at *high* risk of selection bias, which was attributable to the methodology used in most studies to identify the target population. Specifically, if a study population was composed of a convenience sample, reported that a specific study outcome such as self-reported behavioral, emotional, or cognitive symptoms/complaints was an inclusion criterion, or if next of kin, relevant other, or medical practitioner referred or enrolled participants in a study, we rated this domain as *high* risk of bias. Several studies were judged to be at *high* risk of bias due to multiple items within this domain being at *moderate* risk of bias from a lack of reporting (of study recruitment period and participation rates) or from low study participation rates.

**Table 2:** Summary of studies of current athletes.

**First author**	**Year published**	**Primary study site**	**Total *N***	**Played before age 12**	**Age (years)**	**Sample/outcome measures**	**AFE binary vs. continuous**
Houck (90)	2020	University of Florida	3,782	Not reported	Median = 19, IQR = 18–20	NCAA Football Players (CARE)/ Neuropsychological Testing (ImPACT)	Continuous
Brett (84)	2019	Medical College of Wisconsin	1,802	1,249	M = 18; SD = 2	High School and College Football Players from Project Head to Head 1 (PH2H1) and/or Project Head to Head 2 (PH2H2)/ Neuropsychological Testing, Mental Status, Mood, Physical*	Binary
Caccese (87)	2019	University of Delaware	4,376	3,022	*M* = 19; SD = 2	NCAA Football Players and Male Non-contact Athletes (CARE)/Neuropsychological Testing (ImPACT)	Binary
Caccese (123)	2020	University of Delaware	889	694	*M* = 19; SD = 1	US Service Academy Male Cadets Contact and Non-contact Athletes (CARE)/Neuropsychological Testing (ImPACT)	Binary
Caccese (88)	2020	The Ohio State University College of Medicine	1,891 Women; 4,448 Men	Not reported	Women, *M* = 19; SD = 1; Men, *M* = 19; SD = 2	Current NCAA Men and Women Contact and Non-contact Athletes (CARE)/Neuropsychological Testing (ImPACT), Mood (BSI-18), Balance (BESS)	Continuous
Caccese (89)	2020	The Ohio State University College of Medicine	294 evaluated 24–48 h following concussion; 327 evaluated at the time they were asymptomatic	Not reported	M = 19; SD = 1	NCAA Football Players (CARE)/Number of days until asymptomatic, Neuropsychological Testing (ImPACT), Mood (BSI-18), Balance (BESS)	Continuous
Caccese (124)	2020	The Ohio State University College of Medicine	30	Not reported	*M* = 22; SD = 2	College-aged soccer players/Sensorimotor processing: visual, vestibular, and proprioceptive gains and phases	Binary
Asken (111)	2020	University of California, San Francisco	1,570	Not reported	Not reported for football-only sample	NCAA Football Players (CARE)/Wechsler Test of Adult Reading (WTAR) standard score	Continuous

Full summaries of these articles are in the online supplement. NCAA, National Collegiate Athletic Association; CARE, Concussion Assessment, Research and Education Consortium; M, mean; SD, standard deviation; IQR, interquartile range; AFE, age of first exposure; ImPACT, Immediate Post-Concussion Assessment and Cognitive Testing; BSI-18, Brief Symptom Inventory 18; BESS, Balance Error Scoring System. *Outcome Measures included: Immediate Post-Concussion Assessment and Cognitive Testing (ImPACT), Trail Making Test A and B, Wechsler Adult Intelligence Scale−4th Edition (WAIS-IV) symbol search and coding, American College Test (ACT), Standardized Assessment of Concussion (SAC), Sport Concussion Assessment Tool-3rd Edition (SCAT3), Brief Symptom Inventory 18 (BSI-18), Brief Sensation Seeking Scale (BSSS), Disinhibition-11 (DIS-11), Balance Error Scoring System (BESS), King Devick. All studies reported all negative findings (i.e., younger AFE was not associated with worse outcomes).

### Prognostic Factor Measurement

In the ‘prognostic factor measurement' domain, six studies (29%) were at *moderate* risk of bias and fifteen studies (71%) were at *high* risk of bias arising from analyzing and reporting AFE. Studies were considered *moderate* risk of bias if AFE was self-reported by survey or clinical interview without bias limitation techniques rather than determined objectively. Studies were considered *high* risk of bias for a number of reasons, including if AFE was estimated by subtracting current age from self-reported number of years playing the sport or if AFE was analyzed and reported as a dichotomized variable only. Importantly, all studies used self-reported AFE to contact/collision sport—no study ascertained AFE, either retrospectively or prospectively, using electronic health or sporting participation records to objectively verify the start-date of participation in contact/collision sport. Although studies were rated to have a *moderate* risk of bias due to participant self-report of AFE, at this time there is no better measurement method. Even in cases where electronic health or sporting participation registries may exist, it is likely too expensive, obtrusive, or time consuming to obtain. In the case of self-reported data, recall bias is of primary concern; however, there are some solutions to overcome recall bias that we propose for future research. For example, methods to facilitate recall include the use of memory aids and structured screening tests rather than single-item methods (126–129). One specific method that might improve the quality of self-reported AFE is to ask a series of questions, instead of a single question, and to also refer to the participant's grade in school when he started playing football, which will be much easier to remember for many participants compared to their age at that time.

### Outcome Measurement

For the ‘outcome measurement' domain, fifteen studies (71%) used clinically observed outcomes or validated patient-reported outcome measures, and they were at *low* risk of bias. One study (5%) used outcome measures based on clinical opinion of a medical examiner, and it was rated at *moderate* risk of bias. Five studies (24%) were judged to be at *high* risk of bias because reported outcome measures represented only a subset of a larger outcome set and thus may have been selectively reported. Additionally, neuroimaging studies were considered to be at *high* risk of bias if only specific brain regions were examined and reported without a pre-registered study protocol—preceding data collection—being available that specified the *a priori* brain regions of interest.

### Confounding

All studies (*k* = 21, 100%) were considered to be at *high* risk of confounding due to (i) a lack of measuring and reporting potential third variables, and (ii) uncertainty surrounding the type and definition of third variables in included studies. Notably, most studies did not measure or report the following potential third variables: (i) prior concussion history; (ii) diagnosed learning disability/ADHD/learning accommodations; (iii) history of headaches or migraine; (iv) mental health problems; (v) socioeconomic status, race, or ethnicity; (vi) duration of play; or (vii) education, which were deemed to be potentially important third variables in the possible association between AFE and later in life brain health. When those variables were assessed and considered, there was little information provided regarding how the confounding variables were defined, measured, or included in statistical analyses.

Not measuring and adequately adjusting for relevant confounding variables may result in bias toward *or* away from the null—the exact direction and magnitude of this bias is unknown. The role of confounding variables, and other third variables (such as mediating and effect modifying variables), is particularly important when trying to discern the nature of an association, if any, between AFE to contact/collision sport and later in life brain health. Many factors may be associated with both exposure (AFE to tackle football) and outcome (later in life brain health), and therefore may play a pivotal role in understanding the direction and magnitude of this association, irrespective of whether these factors sit on the causal pathway between exposure and outcome. Consequently, it is crucial that future studies investigating this association carefully measure and adjust for potentially important third variables, such as socioeconomic status and concussion history.

### Statistical Analysis/Reporting

Eight studies (38%) were rated at *low* risk of bias. Eleven studies (52%) were rated at *moderate* risk of bias due to the authors performing many comparisons between groups without adjusting for multiple comparisons. It was difficult to determine whether many outcomes and/or analytical approaches were used but only some reported. Without an available pre-registered protocol detailing study outcomes and statistical analysis plans prior to data collection, it was/is not possible to determine selective outcome reporting and selective analytical reporting. One study (5%) was judged to be at *high* risk of bias due to a perceived limitation in the statistical analysis (89), and one study (5%) was considered *high* risk of bias due to clear evidence that data for only a subset of analyses was fully reported, and that the fully reported results were selected based on the (statistically significant) nature of the results (122).

### Risk of Bias Conclusions and Future Recommendations

Our risk of bias assessment of the studies identified that each study had at least two domains at *high* risk of bias, leading to an overall judgement of *high* risk of bias for every study (Table 3). *High* risk of bias ratings were attributable to perceived limitations in the design, conduct, analysis, and/or reporting. Importantly, however, the domain and overall ratings of *high* risk of bias in every study also reflect the stringent nature of risk of bias as a concept.

**Table 3:** Quality in prognosis studies tool ratings (risk for bias ratings).

**First author**	**Year published**	**Retired vs. current athletes**	**Quality in prognosis studies-6 bias domains**						
			**Participation**	**Attrition**	**Prognostic factor measurement**	**Outcome measurement**	**Confounding**	**Statistical analysis/reporting**	**Overall**
Stamm (80)	2015	Retired	High	N/A	High	High	High	Low	High
Stamm (81)	2015	Retired	High	N/A	High	High	High	Low	High
Schultz (82)	2018	Retired	High	N/A	High	High	High	Moderate	High
Kaufmann (83)	2021	Retired	High	N/A	High	High	High	Low	High
Alosco (92)	2017	Retired	High	N/A	Moderate	Low	High	Low	High
Montenigro (93)	2017	Retired	High	N/A	High	Low	High	Low	High
Alosco (94)	2018	Retired	High	N/A	High	Moderate	High	Moderate	High
Solomon (91)	2016	Retired	High	N/A	High	Low	High	Low	High
Roberts (71)	2019	Retired	High	N/A	Moderate	Low	High	Moderate	High
Iverson (86)	2020	Retired	High	N/A	Moderate	Low	High	Moderate	High
Iverson (85)	2021	Retired	High	N/A	Moderate	Low	High	Moderate	High
Bryant (122)	2020	Current & Retired	High	N/A	Moderate	Low	High	High	High
Hunzinger (121)	2021	Current & Retired	High	N/A	Moderate	Low	High	Moderate	High
Houck (90)	2020	Current	Moderate	N/A	High	Low	High	Low	High
Brett (84)	2019	Current	Low	N/A	High	Low	High	Moderate	High
Caccese (87)	2019	Current	Low	N/A	High	Low	High	Moderate	High
Caccese (123)	2020	Current	Moderate	N/A	High	Low	High	Moderate	High
Caccese (88)	2020	Current	Low	N/A	High	Low	High	Moderate	High
Caccese (89)	2020	Current	High	N/A	High	Low	High	High	High
Caccese (124)	2020	Current	High	N/A	High	High	High	Moderate	High
Asken (111)	2020	Current	Low	N/A	High	Low	High	Low	High

*A customized guide for rating these articles is provided in the online supplement. All studies were rated as having an overall high risk for bias*.

Risk of bias is a systematic deviation from the truth in the results of a research study due to limitations in design, conduct, analysis, or reporting, irrespective of what study authors were *capable* of (130–132). For example, the cross-sectional nature of included studies *required* study authors to ascertain AFE using participant retrospective self-report. Participant retrospective self-report can introduce measurement inaccuracies to a study due to recall bias, whereby the accuracy of participants' memories may be influenced by subsequent events and experiences (133–135). In this instance, historical electronic sporting participation records would minimize systematic measurement error of AFE, despite this being nearly impossible for authors to access and use. Therefore, although participants' self-report of their AFE is the most feasible method of prognostic factor measurement, the potential of this method to distort study results from the truth necessitated a *moderate* risk of bias judgement. This is especially notable for studies requiring a binary classification of before or after the age of 12, which represents when subjects in the United States typically were in the 6th or 7th grade. However, future research may consider methods to overcome recall bias, such as including the use of memory aids and structured screening tests rather than single-item methods (126–129).

Studies that provided unclear or ambiguous reports of design, conduct, or analysis were rated as *moderate* risk of bias. This was due to the empirical assertion that suboptimal reporting does not always imply a suboptimal methodology (136). Future research in this area will benefit from transparent pre-specification of study methods, outcomes, and analyses prior to study analyses that enables readers to distinguish between planned and *post-hoc* study decisions and methods.

It is also important for future research to include both planned and independent replication and extension—especially in neuroimaging research where concern has been expressed about a reproducibility or replication “crisis” (137–139)—although these concerns apply to psychology and other fields more broadly where researchers often do not wish to replicate prior work given the pressures and incentives associated with “novel findings” and new discoveries (140, 141). That is, when possible, researchers reporting findings from small clinical and neuroimaging studies should attempt to directly replicate (and also extend) those findings, and independent researchers are also encouraged to attempt to replicate prior studies using the same or similar methods—especially with larger samples. Replication studies with larger, more diverse samples may adjust for potentially confounding factors leading to more generalizable results, despite conflicting findings. Further, future studies should continue to examine the association between AFE and outcomes across the lifespan and with aging.

The dichotomy of views held by investigating research groups may, in itself, be hindering the progress of science toward discovering the true underlying relationship between AFE and midlife and later-life brain health. This could be addressed through so-called “adversarial collaboration” wherein two or more research scientists (or groups) with opposing views work together despite having distinct, competing hypotheses (142–147). By collaborating, those with opposing views hold each other to high standards of scientific design, conduct, analysis, and reporting, resulting in study findings that have greater scientific clarity, certainty, and credibility (given that both research teams have collaboratively undertaken the science producing the observed/published result).

## Discussion

There has been considerable interest in whether earlier AFE to football, and other collision and contact sports, is associated with future problems with brain health. The genesis of this interest was a single small study of retired NFL players (*N* = 42) reporting that those who started playing football before the age of 12 performed worse on two neuropsychological tests and a single-word reading test (80). Furthermore, this study was part of the impetus for tremendous societal interest and legislative advocacy relating to whether tackle football should be banned for youth under a specific age. This first study had methodological limitations, as discussed in this review, and the results from that study have not been replicated by other research groups. The original study might have been exploratory. Exploratory, underpowered studies with considerable methodological and analytical flexibility have an increased risk of observing novel but spurious findings (104, 148, 149). Three follow-up studies have examined neuropsychological test performance in former professional football players and none have found a statistically significant association between earlier AFE and worse cognitive functioning (91–93).

### Clinical Studies of Middle-Aged and Older Adult Men

Beyond the original study, there have been six studies to date examining middle-aged and older adult men who played football to determine if earlier AFE was associated with greater reporting of mental health problems, cognitive deficits, or other neurobehavioral symptoms. Only one study has found such as association using data from subjects recruited into the LEGEND study (*N* = 214) (92). This same research team, however, also using subjects from the LEGEND study and a different methodology, did not find an association between AFE and these same self-reported symptom outcomes (*N* = 93) (93). Four additional independent studies, one large survey of former NFL players (*N* = 3,506) (71), one clinical study of former NFL players (*N* = 45) (91), and two surveys of men who played high school football (*N* = 123, *N* = 186) (85, 86), have not found a statistically significant association between earlier AFE to football and self-reported cognitive, neurobehavioral, or psychological functioning later in life. In one large-scale study of active and retired professional fighters, earlier AFE to combat sport was associated with worse measured cognitive functioning and greater self-reported symptoms of depression and impulsiveness (122).

### Neuroimaging Studies

There have been four studies to date using experimental neuroimaging modalities to examine whether earlier AFE to football is associated with differences in brain macrostructure or microstructure in former football players. Three of those studies have been published by the same research group and they have reported that (i) differences in white matter microstructure (81), as measured by DTI, (ii) differences in thalamic volumes (82), as measured by volumetric analytic methods, and differences in cortical thickness (83) are associated with AFE in former NFL players. These neuroimaging studies used subjects from the DETECT study—researchers should not assume that these are three *independent* studies given that there was likely considerable overlap in subjects across the studies. These findings have not been replicated to date in independent samples of former football players. One independent study of former NFL players did not demonstrate evidence of a detectable association between AFE and neuroimaging outcome measures (91). In a study of combat sport athletes, earlier AFE to fighting was associated with smaller brain structures in some brain regions (122).

### Studies of Current High School and Collegiate Athletes

There have been eight published studies on this topic with current high school and collegiate athletes, which inform possible short- to medium-term associations in current athletes. No published studies of current athletes have reported a statistically significant association between playing football (or other contact sports) before the age of 12 and worse functioning (84, 87, 88, 90, 111, 123, 124), or worse clinical outcome from concussion (89). These large-scale cross-sectional studies of high school and collegiate athletes have found that earlier AFE to football and other contact sports is not significantly associated with worse objectively measured cognitive functioning (84, 87, 88, 90, 123) or greater self-reported physical, cognitive, or emotional symptoms (84, 88). In fact, these five studies (84, 87, 88, 90, 123) examined four independent cohorts amassing nearly 10,000 athletes across 20 different outcome measures, and they did not report a single outcome measure that was worse with earlier AFE to football or other contact sports.

## Conclusions

In summary, the literature on whether earlier AFE to football is associated with later in life cognitive, neurobehavioral, or mental health problems in former NFL players is mixed. In the largest study to date of retired NFL players (*N* = 3,506), there was not a significant association between starting to play football before the age of 12, or earlier AFE analyzed continuously, and worse subjectively-experienced cognitive functioning, depression, or anxiety (71). Smaller studies of former NFL players have shown some associations with neuroimaging findings and clinical outcome variables (80–82, 92, 94). One large study of combat sport athletes does show an association between earlier AFE to professional fighting and smaller brain structures, worse cognitive functioning, and greater subjectively experienced symptoms. Therefore, it is possible that there is an association between AFE to contact/collision sport and brain health in some former professional athletes with very high exposure to repetitive neurotrauma. It will be important to observe whether the findings of prior studies are replicated by future research investigating this association. The best available evidence to date suggests that earlier AFE to contact or collision sports is not associated with worse cognitive functioning or mental health in (i) current high school athletes, (ii) current collegiate athletes (84, 87, 88, 90, 111, 123, 124), or (iii) middle-aged men who played high school football (85, 86).

## Author Contributions

GI conceptualized and designed the review, assisted with conducting the literature review, wrote portions of the manuscript, edited drafts, and agrees to be accountable for the content of the work. FB and JC assisted with conducting the literature review, conducted the risk of bias assessment, wrote portions of the manuscript, edited drafts, and agrees to be accountable for the content of the work. All authors contributed to the article and approved the submitted version.

## Funding

GI acknowledges unrestricted philanthropic support from ImPACT Applications, Inc., the Mooney-Reed Charitable Foundation, the National Rugby League, and the Spaulding Research Institute. These entities were not involved in the study design, interpretation of data, the writing of this article or the decision to submit it for publication.

## Conflict of Interest

GI serves as a scientific advisor for NanoDx^®^, Sway Operations, LLC, and Highmark, Inc. He has a clinical and consulting practice in forensic neuropsychology, including expert testimony, involving individuals who have sustained mild TBIs (including athletes). He has received research funding from several test publishing companies, including ImPACT Applications, Inc., CNS Vital Signs, and Psychological Assessment Resources (PAR, Inc.). He has received research funding as a principal investigator from the National Football League, and subcontract grant funding as a collaborator from the Harvard Integrated Program to Protect and Improve the Health of National Football League Players Association Members. The remaining authors declare that the research was conducted in the absence of any commercial or financial relationships that could be construed as a potential conflict of interest.

## Publisher's Note

All claims expressed in this article are solely those of the authors and do not necessarily represent those of their affiliated organizations, or those of the publisher, the editors and the reviewers. Any product that may be evaluated in this article, or claim that may be made by its manufacturer, is not guaranteed or endorsed by the publisher.
