# Supplementary material for: Age of First Exposure to Contact and Collision Sports and Later in Life Brain Health: A Narrative Review
Source: Front Neurol. 2021 Sep 29;12:727089. doi: 10.3389/fneur.2021.727089 (PMC8511696; doi:10.3389/fneur.2021.727089)
Supplement: Supplementary file 1 [file Data_Sheet_1.pdf]

Online Supplementary Material

Age of First Exposure to Contact and Collision Sports and Later in Life Brain Health: A Narrative Review

Grant L. Iverson, PhD; Fionn Büttner, PhD; & Jaclyn B. Caccese, PhD

Supplement 1: Article Summaries: Purpose of each study and summary of the relevant findings.
Supplement 2: Risk of Bias Coding System
References

Supplement 1, Article Summaries: Purpose of each study and summary of the relevant findings.

Stamm et al. (2015) [1]
Purpose: To examine the association between AFE to football and later-life executive function, memory, and estimated verbal IQ.
Participants: DETECT participants included former NFL players and a control group of former elite noncontact sport athletes, but only the data from the NFL players were included in the analyses. Participants were male, ages 40–69, played at least 2 years in the NFL and 12 years of organized football, and self-reported complaints of cognitive, behavioral, and mood symptoms for at least the last 6 months. The final sample included 42 participants (age range 41–65 years, average age was 52 years).
AFE Definition: AFE was treated as a dichotomous variable and used to divide subjects into two cohorts: before age 12 and age 12 or older (range age 7 to age 17).
Outcome Measures: Wisconsin Card Sorting Test (WCST), Neuropsychological Assessment Battery List Learning test (NAB-LL), Wide Range Achievement Test, 4th edition (WRAT-4), Reading subtest.
Potential confounders examined: Duration of play and education.
Significant Findings: Results of the paired-sample *t* tests suggested mean scores for the WCST % errors, % perseverative responses, % perseverative errors, and % conceptual level responses, NAB-LL immediate recall, and WRAT-4 Reading differed between groups. The AFE<12 group had significantly lower scores than the AFE≥12 group on all outcomes, indicating poorer performance. Results from the mixed-effects linear model and bootstrap analyses suggested all measures of the WCST, NAB-LL, and WRAT-4 Reading tests differed between groups, with the AFE<12 group performing worse than the AFE≥12 group.
Null Findings: Results of the paired-sample *t* tests suggested no differences in mean scores for the WCST % nonperseverative errors, NAB-LL short delay, or NAB-LL long delay.

Stamm et al. (2015) [2]
Purpose: To examine the association between AFE to football and later-life corpus callosum microstructure.
Participants: DETECT participants included former NFL players and a control group of former elite noncontact sport athletes, but only the data from the NFL players were included in the analyses. Participants were male, ages 40–69, played at least 2 years in the NFL and 12 years of organized football, and self-reported complaints of cognitive, behavioral, and mood symptoms for at least the last 6 months. The final sample included 40 participants (age range 40–65 years, average age was 52 years).
AFE Definition: AFE was treated as a dichotomous variable and used to divide subjects into two cohorts: before age 12 and age 12 or older (range age 6 to age 17, median=11.5).
Outcome Measures: Corpus callosum (CC) microstructure using DTI, including fractional anisotropy (FA), trace, axial diffusivity, and radial diffusivity for the whole CC and five sub-regions [i.e., prefrontal (I), premotor and supplementary motor (II), primary motor (III), sensory (IV), and parietal, temporal, and occipital cortical (V)].
Potential confounders examined: Duration of play and body mass index (BMI).
Significant Findings: Results from the mixed-effects linear model suggested the AFE <12 group displayed significantly lower FA in the anterior CC regions (I, II, and III) and higher RD in region I, compared with the AFE≥12 group.
Null Findings: AD and trace did not differ significantly between groups, nor did whole CC FA or RD, FA in regions III/IV, or RD in regions II, III, or IV.

Schultz et al. (2018) [3]
Purpose: To examine the association between AFE to football and thalamic volume.
Participants: DETECT participants included former NFL players and a control group of former elite noncontact sport athletes, but only the data from the NFL players were included in the analyses. Participants were male, ages 40–69, played at least 2 years in the NFL and 12 years of organized football, and self-reported complaints of cognitive, behavioral, and mood symptoms for at least the last 6 months. The final sample included 86 participants (mean age=54.9±7.9 years).
AFE Definition: The age participants started playing organized tackle football was self-reported and treated as a continuous variable (range from 6 to 17 years, mean AFE=11.8±2.6 years).
Outcome Measures: Thalamic volumes (right and left) were quantified from T1-weighted magnetic resonance imaging data. Mood and behavior, psychomotor speed, and visual and verbal memory were also assessed, but were not examined in the context of AFE.
Potential confounders examined: Duration of play, age, BMI, and estimated total intracranial volume.
Significant Findings: Right thalamic volume was associated with AFE after adjusting for total years of play. For every year a participant started playing earlier, the average decrease in thalamic volume was $64.9mm^3$.
Null Findings: There was no association between left thalamic volume and AFE.

Kaufmann et al. (2021) [4]
Purpose: To examine the association between AFE to football and cortical thickness.
Participants: DETECT participants included former NFL players and a control group of former elite noncontact sport athletes. Only data from the former NFL players were included in this study's analyses. Participants were included if they were male, were ages 40–69, played at least 2 years in the NFL at positions known to have extensive head impacts based on helmet accelerometer data (i.e., offensive and defensive linemen, defensive backs, linebackers, running backs), played at least 12 years of organized football, and self-reported complaints of cognitive, behavioral, and mood symptoms for at least the last 6 months. The final sample included 63 participants (mean age=55.5±7.7 years).
AFE Definition: The age participants started playing organized tackle football was self-reported and treated as a continuous variable (min/max values of 6 and 17 years, mean AFE=11.4±2.6 years).
Outcome Measures: Whole-brain analyses of cortical thickness; a cluster-wise Monte Carlo simulation was applied (threshold of 0.05) to adjust for multiple comparisons.
Potential confounders examined: Duration of play, age, BMI, and education (represented by school years).
Significant Findings: A positive statistically significant correlation between AFE and cortical thickness was found in the left parietal cortex (supramarginal gyrus and superior parietal lobule), in the right superior frontal cortex (posterior superior frontal cortex and dorsal precentral gyrus), as well as in the bilateral occipital cortex (cuneal cortex and pericalcarine cortex and lingual gyrus). These findings suggest that the younger a former NFL player began to play football, the thinner the cortex in these brain regions later in life.
Null Findings: There were other clusters without statistically significant correlations between cortical thickness and AFE, but these statistically non-significant associations were not explicitly reported..

Alosco et al. 2017 [5]
Purpose: To examine the association between AFE to football and behavior, mood and cognition.
Participants: The final sample included 214 former football players from the Longitudinal Examination to Gather Evidence of Neurodegenerative Disease (LEGEND) study (mean age=50.68±13.33 years). Inclusion criteria for the LEGEND study are broad to optimize generalizability and include ≥18 years of age and history of participation in an organized sport. The final sample included only male former football players who played high school, college or professional football, and did not participate in any other organized contact sports. Participants with a self-reported history of concussion within 1 year of their initial LEGEND interview were excluded.
AFE Definition: Participants self-reported AFE to football via telephone-administered structured questionnaire. AFE was examined as a dichotomized variable (<12 and ≥12), and as a continuous variable (mean AFE=11.12±2.47 years).
Outcome Measures: Brief Test of Adult Cognition by Telephone (BTACT), Behavior Rating Inventory of Executive Function-Adult Version (BRIEF-A), Center for Epidemiologic Studies Depression Scale (CES-D) and the Apathy Evaluation Scale (AES).
Potential confounders examined: Duration of play, age, years of education, level of play, and history of learning disability.
Significant Findings: Compared with those who began playing football ≥ 12 years of age, those who started playing <12 exhibited higher (i.e., worse) scores on the BRIEF-A, CES-D and the AES. Those with an AFE to football <12 had 2 × (3 × for the CES-D) increased odds for clinically meaningful impaired scores, relative to AFE ≥ 12 on the BRIEF-A, CES-D and the AES. Younger AFE to football was

associated with higher (i.e., worse) scores on the BRIEF-A, CES-D, and the AES. Younger AFE corresponded to increased odds for clinically meaningful impaired scores on the CES-D and AES.
Null Findings: Younger AFE to football, whether analyzed dichotomously at age 12 or continuously, was not associated with worse BTACT scores. Younger AFE was not associated with clinically elevated scores on the BRIEF-A or the BTACT. When history of a learning disability was added to the model, there was loss of significance for the BRIEF-A MI and odds for clinically impaired scores on the CES-D.

Montenigro et al. (2017) [6]
Purpose: To examine the association between RHI exposure and long-term clinical outcomes, and to evaluate the predictive validity of RHI exposure relative to other exposure metrics (i.e., duration of play, AFE to football, concussion history).
Participants: Of the 800 participants in the LEGEND data set at the time of analysis, 93 former amateur football players met the inclusion/exclusion criteria: 1) highest level of football played was at high school or college, 2) no concussion was sustained in the year prior to their initial evaluation, and 3) they did not participate in another contact sport. The final sample included 17 former high school football players and 76 former collegiate football players (mean age=47.3±13.9 years).
AFE Definition: Participants were asked to report their AFE to football; participant's AFE was converted into a dichotomous variable: AFE before age 12 and AFE at age 12 or above (mean AFE=10.9±2.7 years).
Outcome Measures: Brief Test of Adult Cognition by Telephone (BTACT), Behavior Rating Inventory of Executive Function-Adult Version (BRIEF-A), Center for Epidemiologic Studies Depression Scale (CES-D) and the Apathy Evaluation Scale (AES).
Potential confounders examined: Cumulative head impact index (CHII), duration of play, age, and education.
Significant Findings: None noted.
Null Findings: AFE did not add independently to the model, nor did it eliminate the significance of the CHII for predicting clinical outcomes.

Alosco et al. (2018) [7]
Purpose: To examine the association between AFE to football and chronic traumatic encephalopathy (CTE) pathological severity and age of neurobehavioral symptom onset.
Participants: The sample included amateur and professional tackle football players whose brains were donated to the Veteran's Affairs-Boston University-Concussion Legacy Foundation (VA-BU-CLF) Brain Bank as part of the “Understanding Neurologic Injury and Traumatic Encephalopathy (UNITE)” study. Inclusion criteria were broad to optimize generalizability and include a history of RHI exposure; symptomatic status was not part of eligibility criteria. The final sample of brain donors included 211 tackle football players neuropathologically diagnosed with CTE (i.e., CTE+, mean age of death=62.91±17.91 years), some of whom had comorbid neurodegenerative diseases, and 35 football players without CTE (i.e., CTE–, mean age of death=42.17±22.40 years).
AFE Definition: Informants were asked to identify the age the participant began to play organized tackle football. All analyses examined AFE as a continuous variable (CTE+, mean AFE=11.84±2.93 years; CTE-, mean AFE=11.26±2.76 years) and dichotomized into before age 12 and 12 or older.
Outcome Measures: CTE pathological severity (stages I and II vs. stages III and IV), age of cognitive and behavioral/mood symptom onset, variables of AD (Braak stage, CERAD [none/sparse versus moderate/frequent], and ADNC), Lewy body pathology (absence or presence), as

well as the age of functional impairment onset in participants who were determined by the consensus panel to have had ante-mortem dementia.
Potential confounders examined: Duration of play, decade at age 12, age at death, and level of play.
Significant Findings: In the CTE+ participants, younger AFE predicted earlier reported cognitive (2.44 years/year) and behavioral/mood (2.50 years/year) symptom onset, and earlier onset of functional impairment (1.17 years/year) in participants who were determined by a consensus panel to have had ante-mortem dementia. AFE<12 was associated with earlier cognitive and behavioral/mood symptom onset by 13.39 and 13.28 years, respectively. AFE<12 predicted earlier functional impairment onset by 8.02 years. In the CTE-only participants, younger AFE predicted earlier reported cognitive (2.93 years/year) and behavioral/mood (2.66 years/year) symptom onset. AFE<12 was associated with earlier cognitive and behavioral/mood symptom onset by 16.38 and 13.16 years, respectively. AFE<12 predicted earlier functional impairment onset by 5.91 years. In the CTE- cohort, AFE<12 predicted earlier cognitive and behavioral/mood symptom onset by 20.33 and 22.42 years, respectively.
Null Findings: The relationship between AFE (continuous or dichotomized based on age 12) and CTE pathological severity was not significant in the CTE+ or CTE-only participants. In CTE+ participants, AFE to tackle football (continuous or dichotomized based on age 12) was not associated with Braak stage, CERAD score, ADNC, or the presence of Lewy body pathology. In the CTE-only participants, there was no association between AFE and age of functional impairment onset, which the authors attributed to the reduced sample size. In the CTE- cohort, relationships between AFE (continuous) and age of neurobehavioral symptom onset was not significant, which again, the authors attributed to the small sample size.

Solomon et al. (2016) [8]
Purpose: To examine the association between years of exposure to pre–high school football (PreYOE) and neuroradiological, neurological, and neuropsychological outcome measures.
Participants: Former NFL players recruited through the NFL Players Association, who were ≤60 years old and with no history of brain surgery; brain tumors, strokes, multiple sclerosis, or seizures that began before entering the NFL (except febrile seizures); HIV or AIDS; significant head injuries from automobile accidents or other nonathletic trauma to the head; concussions/mild traumatic brain injuries (mTBIs) after the NFL career with minutes of loss of consciousness or hospitalization; open heart surgery, organ transplant surgery, or carotid artery surgery; treatment with chemotherapy or radiation therapy for cancer affecting the brain or spinal cord; renal failure requiring dialysis or liver failure, resulting in cirrhosis or request for liver transplant; significant alcohol abuse and/or drug abuse in the past or present, manifested by having been suspended by a league, arrested for driving under the influence, or treated in a rehabilitation facility for drug or alcohol abuse; daily use of an illegal drug; and daily intake of more than 4 beers or more than 2 “hard liquor” drinks per day during the past 5 years. The final sample included 45 participants (mean age=46.7±9.1 years).
AFE Definition: Participants self-reported years of pre–high school football participation (mean number of years of pre–high school football=2.5±2.3).
Outcome Measures: Presence or absence of microbleeds (from susceptibility-weighted imaging), global white matter FA (from DTI), presence or absence of a small or large cavum septum pellucidum (from MRI), Beck Depression Inventory-2nd Edition (BDI-II), Patient Health Questionnaire (PHQ), Mental Status: Mini-Mental State Evaluation (MMSE), Test of Memory Malingering (TOMM), Brief Visuospatial Memory Test–Revised (BVMT-R), California Verbal Learning Test–2nd Edition (CVLT-II), Trail Making Test A and B,

Wechsler Adult Intelligence Scale–3rd Edition (WAIS-III) digit symbol and letter number sequencing subtests, Controlled Word Association Test (COWAT), Category Fluency (animals), Wechsler Test of Adult Reading–IQ (WTAR-IQ), and Immediate Post-Concussion Assessment and Cognitive Testing (ImPACT).
Potential confounders examined: Age, BMI, learning disability, years of NFL experience, number of NFL concussions, and position.
Significant Findings: None noted.
Null Findings: None of these outcome measures was associated with pre–high school football years of exposure.

Roberts et al. (2019) [9]
Purpose: To examine the association between seasons of play and playing position in the NFL with cognition-related quality of life (QOL), depression, and anxiety, and to examine the associations between self-reported concussion symptoms during playing years, non-head-related injuries, and AFE to football with the outcomes.
Participants: Former players who participated in the NFL since 1960 recruited through the NFL Players Association (n=3,506, mean age=52.8±14.2 years).
AFE Definition: Players were asked, "How old were you when you began to play organized football?"; AFE was dichotomized as <12 or ≥12 years and examined as a continuous variable (mean AFE=11.8±3.1 years).
Outcome Measures: Cognition-Related Quality of Life (QOL), Depression or anxiety as determined by Patient Health Questionnaire-4 (PHQ-4) and current use of medication for depression or anxiety
Potential confounders examined: Age at interview and race.
Significant Findings: None noted.
Null Findings: AFE to football was not associated with cognition-related QOL, depression, or anxiety, whether continuously or dichotomized at <12 years.

Iverson et al. (2020) [10]
Purpose: To examine the association between AFE to football and mid-life brain health.
Participants: 435 men, ages 35-55 years, were recruited via an online crowdsourcing platform (Amazon Mechanical Turk [mTurk]. The final sample included 123 men, who played high school and/or college football (mean age=44.82±6.19 years).
AFE Definition: Participants were asked to self-report the age they began playing football; participants were stratified into two groups: those who reported starting football prior to 12 years of age and those who reported starting football at age 12 or older (mean AFE=11.2±2.8 years). AFE to football was also examined as a continuous variable in the context of PHQ-8 and BC-PSI scores.
Outcome Measures: Patient Health Questionnaire – 8 (PHQ-8), British Columbia Post-Concussion Symptom Inventory (BC-PSI), and a survey of medical history (including current medication history) and concussion history.
Potential confounders examined: Total years of football participation.
Significant Findings: Those reporting earlier football exposure reported a greater number of lifetime concussions (mean=2.7±3.0) than those who reported starting football at age 12 or later (mean=1.9±2.3). Individuals who started playing football before age 12 reported sustaining their first concussion at a younger age than those who started at 12 years or older (AFE <12, mean=14.3±4.9 years; AFE ≥12, mean = 15.6±4.3 years).

**Null Findings:** The groups did not differ in the proportion of participants who were prescribed medications for anxiety, depression, memory loss, chronic pain, or headaches. Moreover, there was no significant difference in their lifetime history of treatment by a mental health professional. There were no significant differences between groups in their ratings of depression, anxiety, anger, concentration problems, memory problems, headaches, migraines, neck or back pain, or chronic pain over the past year. The groups did not differ significantly on the PHQ-8 or BC-PSI total scores. There was no significant correlation between AFE to football, as a continuous variable, and PHQ-8 or BC-PSI scores.
**Iverson et al. (2021)** [11]<br>**Purpose:** To examine the association between AFE to football and brain health in middle-aged and older adult men.<br>**Participants:** Men, over age 35 years, were recruited via an online crowdsourcing platform [Amazon Mechanical Turk (mTurk)]. The final sample included 186 men who played high school and/or college football (mean age=51.78±10.93 years).<br>**AFE Definition:** Participants were asked to self-report the age they began playing football; participants were stratified into two groups: those who reported starting football prior to 12 years of age and those who reported starting football at age 12 or older (overall mean AFE=11.42±2.98 years). AFE to football was also examined as a continuous variable in the context of PHQ-8 and BC-PSI scores.<br>**Outcome Measures**: Patient Health Questionnaire-8 (PHQ-8), British Columbia Post-Concussion Symptom Inventory (BC-PSI), and a survey of medical history (including current medication history) and concussion history.<br>**Potential confounders examined:** Total years of football participation.<br>**Significant Findings:** Those reporting earlier football exposure reported a greater number of lifetime concussions (mean=1.95±1.79) than those who reported starting football at age 12 or later (mean=1.28±1.52). Individuals who started playing football before age 12 reported sustaining their first concussion at a younger age than those who started at 12 years or older (AFE <12, mean=16.91±10.17 years; AFE ≥12, mean = 17.59±8.78 years).<br>**Null Findings:** There was a similar proportion of men in both groups who reported that they had been prescribed medications for depression, anxiety, chronic pain, headaches, or memory loss. There was no between-group difference in lifetime history of treatment by a mental health professional. There was a similar proportion of men in both groups who reported being diagnosed with stroke, Parkinson’s disease, dementia, arthritis, sleep apnea, heart attack, and cancer. A similar proportion of men in both groups endorsed significant problems with mental health, cognitive functioning, headaches, and chronic pain, over the past year and over the past week. There were no significant differences between groups in their ratings of depression, anger, anxiety, headaches, migraines, neck or back pain, chronic pain, concentration problems, or memory problems over the past week or the past year. The two AFE groups did not differ significantly on PHQ-8 or BC-PSI scores. Similarly, the two AFE groups did not differ in the proportion of the sample who had a total PHQ-8 score of 10 or more. There was no correlation between AFE to football, as a continuous variable, and PHQ-8 or BC-PSI scores.
**Bryant et al. (2020) [12]**<br>**Purpose:** To examine the association between AFE to fighting sports and brain structure, cognitive performance, and clinical neuropsychiatric symptoms.<br>**Participants:** Participants included current and retired professional fighters (boxers, mixed martial artists, and martial artists). Retired fighters had to have had at least 10 professional fights. Inclusion criteria were being at least 18 years of age, having at least a fourth-grade

reading level, fluency in English or Spanish, and the ability to undergo 3-tesla MRI of the brain. The final sample included 442 active fighters (mean age=29.05±5.44 years) and 64 retired fighters (mean age=47.80±9.53 years).
AFE Definition: AFE to competitive fighting was defined as the study participant's self-reported age (in years) when competitive fighting began. This age was the earlier of either amateur or professional competitive fighting experience.
Outcome Measures: Hippocampus, amygdala, caudate, putamen, thalamus, and corpus callosum volume, CNS Vital Signs, iComet C3 (BESS, Trails A, Trails B, Processing Speed, Simple Reaction Time, and Choice Reaction Time), PHQ-9, Barratt Impulsiveness Scale version 11 (BIS-11).
Potential confounders examined: Fighter type, age, education in years, ethnicity, number of fights, years of professional fighting, and intracranial volume.
Significant Findings: Brain MRI data showed that there were significant correlations between earlier AFE and smaller hippocampal volume for both retired and active fighters on both the left and right sides. Similarly, there was a significant correlation between earlier AFE and smaller posterior corpus callosum volume for both cohorts. Active fighters showed a correlation between earlier AFE and smaller left amygdala volume. For the right amygdala, there was a correlation between earlier AFE and smaller volume for retired fighters. On the CNS Vital Signs clinical report, earlier AFE in active fighters significantly correlated with decreased processing speed and decreased psychomotor speed. Retired fighters showed a correlation between earlier AFE and higher BESS score. Active fighters showed a correlation between earlier AFE and decreased processing speed. On the PHQ-9 screen for depressive symptoms, retired fighters showed a correlation between earlier AFE and higher PHQ-9 score. On the BIS-11, retired fighters exhibited a correlation between earlier AFE and higher total BIS score. Retired fighters demonstrated correlations between earlier AFE and worse attentional, motor, and non-planning scores.
Null Findings: The MRI data showed no significant correlations between AFE and thalamus, caudate, or putamen volume on either side in either cohort. Likewise, there was no correlation between anterior or central corpus callosum volume and AFE in either cohort. For the left amygdala, there was no correlation between AFE and volume for retired fighters. For the right amygdala, there was no correlation between AFE and volume for active fighters. There were no correlations between AFE and verbal memory or reaction time for the active fighters. The CNS Vital Signs yielded no significant correlations for retired fighters. There was no correlation between AFE and Balance Error Scoring System (BESS) score for active fighters. Retired fighters showed no correlation between AFE and processing speed. There were no correlations between AFE and Trails A, Trails B, simple reaction time, or choice reaction time scores for either retired or active fighters. There was no correlation between AFE and PHQ-9 score for active fighters. There was no correlation between AFE and BIS-11 score for active fighters.

Hunzinger et al. (2021) [13]
Purpose: To examine the association between AFE to RHI through contact/collision sports and self-reported outcomes.
Participants: Current and former rugby players aged 18 years or older with at least one year of contact rugby playing experience were recruited via rugby-specific forums on social media (e.g., Reddit, Facebook). The final sample included 1,037 community rugby players (mean age=31.6±11.3 years, 59% men).
AFE Definition: Participants self-reported AFE to various contact and collision sports, including boxing, American football, ice hockey, lacrosse, rugby, soccer, and wrestling. AFE was examined both continuously (women, mean AFE=10.0±5.6 years; men, mean AFE=8.7±4.2 years) and dichotomously at age 12.
Outcome Measures: Brief-Symptoms Inventory 18, Short Form Health Survey 12, and Satisfaction with Life Scale.
Potential confounders examined: Age, cumulative seasons of contact/collision sports, and concussion history. Sex-specific analyses were used.
Significant Findings: None noted.
Null Findings: Findings from the generalized linear models suggested that AFE to contact/collision sports was not a significant predictor for any of the outcomes among women or among men. Furthermore, there were no significant differences between AFE <12 and AFE ≥12 groups for any of the outcomes among women or among men.

Houck et al. (2020) [14]
Purpose: To examine the association between sociodemographic factors, estimated head impact exposure, including AFE to football, and academic aptitude on ImPACT scores.
Participants: Data were obtained from the National Collegiate Athletic Association (NCAA)-Department of Defense (DoD) Concussion Assessment, Research, and Education (CARE) Consortium. The CARE consortium consists of 26 colleges and universities and four US service academies. The final sample for football-specific analyses included 3,782 football players (median age=19 years).
AFE Definition: AFE was calculated by subtracting the total years of football participation from the participant's current age. Therefore, AFE assumed that the participant played football continuously from when they first began (median age=9 years).
Outcome Measures: Immediate Post-Concussion Assessment and Testing (ImPACT) Verbal Memory, Visual Memory, Visual Motor Speed, and Reaction Time.
Potential confounders examined: Academic aptitude (i.e., z-transformed SAT/ACT scores as a surrogate measure of educational quality), race, socioeconomic status (SES), concussion history, history of ADHD/LD, and psychological distress (i.e., Brief Symptom Inventory – 18 Item [BSI-18]).
Significant Findings: Later AFE to football was associated with better academic aptitude (r=0.077).
Null Findings: There was no relationship between AFE and test performance on any ImPACT composite scores.

**Brett et al. (2019) [15]**<br>**Purpose:** To examine the association between AFE to football and intermediate (ie, in adolescence/young adulthood) behavioral, cognitive, emotional/psychological, and physical outcomes.<br>**Participants:** Participants were football players from 9 high schools and 4 colleges in southeastern Wisconsin enrolled in the prospective Project Head to Head 1 (PH2H1) and/or Project Head to Head 2 (PH2H2) studies between August 2012 and September 2017. To maintain generalizability, exclusion criteria involved only 2 factors, invalid performance on computerized neurocognitive tests (CNT) and those in which AFE could not be obtained (i.e., age or years of participation was not provided). The final sample included 1,802 football players for analysis (mean age= 17.99 ± 1.83 years)<br>**AFE Definition:** AFE was was derived from participants' response to questions about the number of years participating in the sport and current age (i.e., years of participation subtracted from age). Following precedent from prior literature, AFE was dichotomized into two groups: AFE before the age of 12 years and at/after the age of 12 years (mean AFE=10.11±2.96 years).<br>**Outcome Measures**: Immediate Post-Concussion Assessment and Cognitive Testing (ImPACT) Verbal Memory, Visual Memory, Visual Motor Speed, and Reaction Time, Trail Making Test A and B, Wechsler Adult Intelligence Scale–4th Edition (WAIS-IV) symbol search and coding, American College Test (ACT), Standardized Assessment of Concussion (SAC), Sport Concussion Assessment Tool-3rd Edition (SCAT3), Brief Symptom Inventory 18 (BSI-18), Brief Sensation Seeking Scale (BSSS), Disinhibition-11 (DIS-11), Balance Error Scoring System (BESS), King Devick.<br>**Potential confounders examined:** Age, height, weight, race, ADHD, LD, IEP, ASD, meningitis, balance disorder, seizure disorder, vision problems, hearing problems, stroke, diabetes, psychiatric diagnosis, migraine disorder, headache disorder, sleep disorder, SES, self-reported concussion history, number of hours of sleep, alcohol and prescription medicine use, WTAR score, and position were examined. Age, race, weight, a diagnosis of ADHD, household socioeconomic status (SES), number of diagnosed sports-related concussions (SRCs), number of undiagnosed SRCs, and typical hours of sleep per night were included in the models**.**<br>**Significant Findings:** Those who began playing football before the age of 12 years exhibited significantly higher (better) visual memory scores, and lower (better) somatization symptom endorsement<br>**Null Findings:** There were no group differences in Verbal Memory, Visual Motor Speed, and Reaction Time, Trail Making Test A and B, WAIS-IV symbol search or coding, ACT, SAC, SCAT3, BSI-18 anxiety or depression, BSSS, DIS-11, BESS, or King Devick scores.
**Caccese et al. (2019) [16]**<br>**Purpose:** To examine the association between AFE to football and neurocognitive performance and symptom severity scores.<br>**Participants:** Data were obtained from the National Collegiate Athletic Association (NCAA)-Department of Defense (DoD) Concussion Assessment, Research, and Education (CARE) Consortium. The CARE consortium consists of 26 colleges and universities and four US service academies. The final sample included 4,376 men [football (n = 3,462), non-contact (n = 914); mean age=19.3±1.5 years].<br>**AFE Definition:** AFE was calculated by subtracting the total years of football participation from the participant's current age. Therefore, AFE assumed that the participant played football continuously from when they first began. AFE was used to divide participants into two cohorts: AFE < 12 and AFE ≥ 12.<br>**Outcome Measures**: Immediate Post-Concussion Assessment and Testing (ImPACT) Verbal Memory, Visual Memory, Visual Motor Speed, Reaction Time, and Symptom Severity.

**Potential confounders examined:** Learning accommodation status, number of previous concussions, and age.<br>**Significant Findings:** The group-by-AFE was a significant predictor of symptom severity scores; pairwise comparisons suggested that football, AFE < 12 interaction reported lower severity scores than all other groups, and that football, AFE ≥ 12 interaction reported lower symptom severity scores than non-contact, AFE < 12.<br>**Null Findings:** The group-by-AFE interaction was not significant for Verbal Memory, Visual Memory, Visual Motor Speed, or Reaction Time composite scores.
**Caccese et al. (2020) [17]**<br>**Purpose:** To examine the association between AFE to contact sports participation and neurocognitive performance and symptom severity scores.<br>**Participants:** Data were obtained from the National Collegiate Athletic Association (NCAA)-Department of Defense (DoD) Concussion Assessment, Research, and Education (CARE) Consortium. The CARE consortium consists of 26 colleges and universities and four US service academies. The final sample included 891 male cadets from the U.S. service academies in contact sports (i.e., varsity ice hockey [n = 81], soccer [n = 119], wrestling [n = 170], rugby [n = 10], and lacrosse [n = 211]) or non-contact sports (n = 298); mean age=19±1 years.<br>**AFE Definition:** AFE was calculated by subtracting the number of years the participant reported playing contact or non-contact sports from the participant's current age. Therefore, AFE assumed that the participant played the sport continuously from when they first began. AFE was used to divide participants into two cohorts: AFE < 12 and AFE ≥ 12.<br>**Outcome Measures**: Immediate Post-Concussion Assessment and Testing (ImPACT) Verbal Memory, Visual Memory, Visual Motor Speed, Reaction Time, and Symptom Severity.<br>**Potential confounders examined:** Learning accommodation status, concussion history, and age.<br>**Significant Findings:** None noted.<br>**Null Findings:** The group-by-AFE interaction was not significant for any of the ImPACT composite scores.
**Caccese et al. (2020) [18]**<br>**Purpose:** To examine the association between AFE to RHI and neurocognitive performance, psychological distress, and postural stability.<br>**Participants:** Data were obtained from the National Collegiate Athletic Association (NCAA)-Department of Defense (DoD) Concussion Assessment, Research, and Education (CARE) Consortium. The CARE consortium consists of 26 colleges and universities and four US service academies. The final sample included 1,891 women (1,056 in contact sports, 712 in non-contact sports; mean age=18.9±1.2 years, mean AFE=7.7 ± 3.2 years) and 4,448 men (1,095 in contact sports, 2,653 in football, 363 in non-contact sports; mean age=19.3±1.5 years, mean AFE=8.6±3.2 years).<br>**AFE Definition:** AFE (continuous) was defined as the participant's age at the time of assessment minus the number of years the participant reported playing his/her primary sport. Therefore, AFE assumed that the participant played the sport continuously from when they first began.<br>**Outcome Measures**: Immediate Post-Concussion Assessment and Cognitive Testing (ImPACT), Brief Symptom Inventory 18 (BSI-18), Balance Error Scoring System (BESS). Outcomes were modeled independently for men and women.<br>**Potential confounders examined:** Concussion history, neurodevelopmental history, migraine history, socioeconomic status, race/ethnicity.

**Significant Findings:** Earlier AFE to contact sports was associated with higher (better) Verbal Memory and Visual Memory composite scores. Earlier AFE to football was associated with lower (better) Depression and Global Severity Index sub-scores and PCSS Symptom Severity scores.<br>**Null Findings:** There was no association between earlier AFE to RHI exposure and worse neurocognitive performance, greater psychological distress, or worse postural stability for men or for women.
**Caccese et al. (2020) [19]**<br>**Purpose:** To examine the association between AFE to football and clinical measures throughout recovery following concussion.<br>**Participants:** Data were obtained from the National Collegiate Athletic Association (NCAA)-Department of Defense (DoD) Concussion Assessment, Research, and Education (CARE) Consortium. The CARE consortium consists of 26 colleges and universities and four US service academies. The final analyses included an overlapping sample of 294 NCAA football players (mean age=19±1 years) evaluated 24–48 hours following concussion and 327 (mean age=19±1 years) evaluated at the time they were initiated their return-to-play progression.<br>**AFE Definition:** AFE was defined as the participant's age at the time of baseline assessment minus the number of years the participant reported playing football. Therefore, AFE assumed that the participant played football continuously from when they first began.<br>**Outcome Measures**: Days until they initiated their return-to-play progression, Immediate Post-Concussion Assessment and Cognitive Testing (ImPACT), Brief Symptom Inventory 18 (BSI-18), Balance Error Scoring System (BESS). For all outcome measures change scores (i.e., post-injury score – baseline score) were analyzed.<br>**Potential confounders examined:** Race/ethnicity, socioeconomic status, concussion history, neurodevelopmental history, psychiatric history, history of depression, migraine history, headache history, setting (Competition or Practice/Training), LOC, PTA, RGA, delayed removal from play and/or additional head trauma, and initial post-injury (<6 hours) symptom severity.<br>**Significant Findings:** In unadjusted regression models, younger AFE was associated with lower (worse) ImPACT Visual Motor Speed (R^2=0.031) at 24-48 hours following injury and lower (better) BSI-18 Somatization sub-scores (R^2=0.014) when the athletes initiated their return-to-play progression. Results of the adjusted models were the same as the results of the unadjusted models. Effect sizes were small (i.e., ImPACT Visual Motor Speed composite score, f^2=0.036; BSI-18 Somatization sub-score, f^2=0.014).<br>**Null Findings:** AFE was not associated with the number of days until asymptomatic, other ImPACT composite scores, BESS total score, or other BSI-18 sub-scores.
**Caccese et al. (2020) [20]**<br>**Purpose:** To compare sensory reweighting for upright stance between college-aged soccer players who began soccer heading ages 10 years and younger (AFE ≤ 10) and those who began soccer heading after age 10 (AFE > 10).<br>**Participants:** Potential participants included both men and women between ages 18 and 30 years, who were current members of a soccer team (i. e., varsity, club, intramural). Exclusionary criteria included any head, neck, or lower extremity injury in the past six months; history of vestibular or ocular dysfunction; currently taking any medications affecting balance; history of any neurological disorders (e. g., seizure disorders); unstable cardiac or pulmonary disease; goalkeepers. The final sample included 30 soccer players (14 women, 16 men; mean age=22±2 years).<br>**AFE Definition:** Self-reported age of first exposure to soccer heading.

**Outcome Measures**: Gain and phase of center-of-mass (COM) relative to the visual stimulus, to the galvanic vestibular stimulation stimulus, and to the vibration stimulus, COM 95% area, and COM sway velocity.<br>**Potential confounders examined:** Years of soccer participation.<br>**Significant Findings:** None noted.<br>**Null Findings:** There were no differences (i. e., unadjusted and adjusted models) in gains or phases to any modality, COM 95% area, or COM sway velocity between groups.
**Asken et al. (2020) [21]**<br>**Purpose:** To examine the association between concussion history and cumulative exposure to collision sports, including AFE, with word-reading skills, and to evaluate how head trauma history compares to other known predictors of word-reading and cognitive test scores, such as SES, race, learning disorder (LD) diagnosis, and academic aptitude.<br>**Participants:** Data were obtained from the National Collegiate Athletic Association (NCAA)-Department of Defense (DoD) Concussion Assessment, Research, and Education (CARE) Consortium. The CARE consortium consists of 26 colleges and universities and four US service academies. The final analyses included 1,570 NCAA football players.<br>**AFE Definition:** AFE was defined as the participant's age at the time of baseline assessment minus the number of years the participant reported playing football. Therefore, AFE assumed that the participant played football continuously from when they first began.<br>**Outcome Measures**: Wechsler Test of Adult Reading (WTAR).<br>**Potential confounders examined:** Concussion history, cumulative years of exposure to collision sports, race, socioeconomic status, SAT/ACT scores, and learning disorder.<br>**Significant Findings:** None noted.<br>**Null Findings:** There was no effect of AFE to football on WTAR standard score.

Note: CTE = Chronic traumatic encephalopathy, NFL = National Football League, NCAA = National Collegiate Athletic Association, DETECT = Diagnosing and Evaluating Traumatic Encephalopathy Using Clinical Tests, LEGEND = Longitudinal Examination to Gather Evidence of Neurodegenerative Disease, UNITE = Understanding Neurologic Injury and Traumatic Encephalopathy, CARE = Concussion Assessment, Research and Education, M = mean, SE = standard error, SD = standard deviation, IQR = interquartile range, AFE = age of first exposure, CERAD = Consortium to Establish a Registry for Alzheimer's Disease, ADNC = Alzheimer's disease neuropathologic change, SWI = susceptibility weighted imaging, DTI = diffusion tensor imaging, MRI = magnetic resonance imaging.

Supplement 2: Risk of Bias Coding System

Age of First Exposure to Contact and Collision Sports and Later-in-Life Brain Health Quality in Prognostic Studies (QUIPS) Tool

1 Review question

Is there an association between earlier age of first exposure (AFE) to contact or collision sports and symptom reporting, cognitive functioning, other clinical measures, and/or neuroimaging findings?

2 Study Participation: To judge the risk of selection bias (likelihood that relationship between prognostic factor, AFE, and outcome is different for participants and eligible non-participants).

2.1 Source of target population - The source population or population of interest is adequately described for key characteristics.

- Consider *low risk* of bias if study population of interest is completely described including relevant participant demographic information such as sex, age (range), sporting type (including contact/noncontact), level of play, and if the demographic information of the recruited population reflects the study population of interest.
- Consider *moderate risk* of bias if the study population of interest is incompletely described.
- Consider *high risk* of bias if the demographic information of the recruited population does not completely reflect the study's target population of interest.

2.2 Method used to identify population - The sampling frame and recruitment are adequately described, including methods to identify the sample sufficient to limit potential bias (number and type used, e.g., referral patterns in health care).

- Consider *low risk* of bias if the target population is identified using distinctive participant characteristics relevant to the study population of interest (e.g., sex, age, sporting type, and level of play).
- Consider *moderate risk* of bias if a study population is retrospectively identified using membership of exclusive cohort within the study population (e.g., NFL Players' Association).
- Consider *high risk* of bias if a study population is composed of a convenience sample.
- Consider *high risk* of bias if the target population is not identified using important participant characteristics relevant to the study population of interest.
- Consider *high risk* of bias if reporting a study outcome (or closely related surrogate outcome), such as self-reported behavioral, emotional, or cognitive symptoms/complaints, is a criterion for inclusion in the study.
- Consider *high risk* of bias if next of kin, relevant other, or medical practitioner refers or enrolls participants in study.

2.3 Recruitment period - Recruitment period is adequately described.

- Consider *low risk* of bias if including recruitment start-date (year) and recruitment end-date (year).
- Consider *moderate risk* of bias if no information, or a lack of information, is reported by study authors about the study recruitment period.
- Consider *high risk* of bias if recruitment period is unrealistically short (e.g., < 1 year) or excessively long (e.g., >5 years). For example, a very short recruitment period in a cross-sectional study, in which participants may be recruited based on very specific criteria, such as clinical presentation, may introduce selection bias by recruiting a greater proportion of participants who are very symptomatic and therefore enthusiastic to be involved (due to the perceived benefit of being involved in the study) and may therefore volunteer immediately. Conversely, an excessively long recruitment period may introduce selection bias by enrolling participants who are not initially interested in participating in the study but develop symptoms throughout the recruitment period and become interested in participating in the study due to their knowledge of the study's purpose (and perceived benefits of being involved in the study).

2.4 Place of recruitment - Place of recruitment (setting and geographic location) are adequately described.

- Consider *low risk* of bias if recruitment site is specified (e.g., collegiate or high school athletic setting, retired athlete cohort recruited/contacted via (e)mail/telephone).
- Consider *moderate risk* of bias if no information, or a lack of information, is reported by study authors about the recruitment site.
- Consider *high risk* of bias if differential recruitment sites exist between groups with different levels of the prognostic factor (e.g., AFE <X years and AFE ≥X years), which may introduce systematic differences between groups relating to the outcome variable.

2.5 Inclusion & exclusion criteria - Inclusion and exclusion criteria are adequately described (e.g., including explicit diagnostic criteria description).

- Consider *low risk* of bias if inclusion/exclusion criteria are adequately described and are equal across all groups with different levels of the prognostic factor [not including, e.g., Contact, AFE <X years; Noncontact, AFE <X years].
- Consider *moderate risk* of bias if no information, or a lack of information, regarding inclusion/exclusion criteria are reported.
- Consider *high risk* of bias if inclusion/exclusion criteria are different across groups with different levels of the prognostic factor.

2.6 Adequate study participation - There is adequate participation in the study by eligible individuals.

- Consider *low risk* of bias if > 80% study participation/response from identified/eligible cohort.
- Consider *moderate risk* of bias if 60-80% study participation/response from identified/eligible cohort.
- Consider *moderate risk* of bias if study participation/participant response is not reported.
- Consider *high risk* of bias if < 60% study participation/response from identified/eligible cohort.

2.7 Baseline characteristics - The baseline study sample (i.e., individuals entering the study) is adequately described for key characteristics.

- Consider *low risk* of bias if there is complete measurement and reporting of important participant demographic characteristics, such as sex, age, sporting, and level of play.
- Consider *moderate risk* of bias if some, but not all, relevant participant demographic information is assessed.
- Consider *high risk* of bias if important participant demographic characteristics are not assessed or considered.

2.8 Summary of study participation - The study sample represents the population of interest on key characteristics, sufficient to limit potential bias of the observed relationship between the prognostic factor (AFE) and outcome.

3 Study Attrition: To judge the risk of attrition bias (likelihood that relationship between prognostic factor, AFE, and outcome are different for completing and non-completing participants).

Due to the cross-sectional nature of the vast majority of included studies, we did not consider this subdomain.

3.1 Proportion of baseline sample available for analysis - Response rate (i.e., proportion of study sample completing the study and providing outcome data) is adequate.

3.2 Attempts to collect information on participants who dropped out - Attempts to collect information on participants who dropped out of the study are described.

3.3 Reasons and potential impact of subjects lost to follow-up - Reasons for loss to follow-up are provided.

3.4 Outcome and prognostic factor information of those lost to follow-up - There are no important differences between key characteristics and outcomes in participants who completed the study and those who did not.

3.5 Study Attrition Summary - Loss to follow-up (from baseline sample to study population analyzed) is not associated with key characteristics (i.e., the study data adequately represent the sample) sufficient to limit potential bias to the observed relationship between the prognostic factor (AFE) and outcome.

4 Prognostic Factor Measurement: To judge the risk of measurement bias related to how the prognostic factor, AFE, was measured (e.g., differential measurement of prognostic factor related to the level of outcome).

4.1 Definition of the Prognostic Factor - A clear definition or description of prognostic factor is provided.

- Consider *low risk* of bias if age of first exposure is defined as the athlete’s age of first participation in sport.
- Consider *moderate risk* of bias if the definition of age of first exposure is not reported.

- Consider *high risk* of bias if years of sporting participation are used instead of age of first exposure (calculated or directly reported).

4.2 Valid and Reliable Measurement of Prognostic Factor - Method of prognostic factor measurement is adequately valid and reliable to limit misclassification bias (e.g., may include relevant outside sources of information on measurement properties, also characteristics, such as blind measurement and limited reliance on recall).

- Consider *low risk* of bias if the age of first exposure is determined, either retrospectively or prospectively, using electronic health or sporting participation records that objectively verify the start-date of participation in (contact/collision) sport.
- Consider *low risk* of bias if study investigator introduced bias limitation techniques by identifying potentially unreliable reports of age of first exposure and excluding the relevant participants.
- Consider *low risk* of bias if age of first exposure is analyzed and reported as a continuous variable or analyzed and reported as both a continuous and dichotomized variable.
- Consider *moderate risk* of bias if the age of first exposure is self-reported by survey or clinical interview.
- Consider *moderate risk* of bias if no information, or a lack of information, about the method used to measure age of first exposure is reported.
- Consider *high risk* of bias if the age of first exposure is estimated by subtracting current age from self-reported number of years playing the sport (for current athletes).
- Consider *high risk* of bias if age of first exposure is self-reported by subjects who have known cognitive deficits, such as mild cognitive impairment or dementia.
- Consider *high risk* of bias if an informant, such as a spouse, reports the age of first exposure, instead of the participant himself/herself.
- Consider *high risk* of bias if age of first exposure is analyzed and reported as a dichotomized variable only, with only theoretical, rather than empirical, rationale.

4.3 Method and setting of prognostic factor (AFE) measurement - The method and setting of measurement of the prognostic factor is the same for all study participants.

- Consider *low risk* of bias if method and setting of prognostic factor measurement is comparable for all study participants.
- Consider *moderate risk* of bias if no information, or little information, about the method and setting of prognostic factor measurement is reported by study authors.
- Consider *high risk* of bias if method and setting of prognostic factor measurement is different between groups with different levels of the prognostic factor (e.g., AFE $<$X years and AFE $\geq$X years).

4.4 Proportion of data on prognostic factor available for analysis - Adequate proportion of the study sample has complete data for the prognostic factor, AFE, variable.

- Consider *low risk* of bias if >80% prognostic factor data is available for analysis.
- Consider *moderate risk* of bias if 60%-80% prognostic factor data is available for analysis.

- Consider *moderate risk* of bias if missing prognostic factor (AFE) information is not reported.
- Consider *high risk* of bias if <60% prognostic factor data is available for analysis.

4.5 Method used for missing data - Appropriate methods of imputation are used for missing prognostic factor (AFE) data.

Participants without prognostic factor (AFE) are excluded from most studies, so we did not consider this subdomain.

4.6 Prognostic Factor (AFE) Measurement Summary – Prognostic factor is adequately measured in study participants to sufficiently limit potential bias.

5 Outcome Measurement: To judge the risk of bias related to the measurement of outcome (e.g., differential measurement of outcome related to the baseline level of the prognostic factor).

5.1 Definition of the Outcome - A clear definition of outcome is provided, including duration of follow-up and level and extent of the outcome construct.

- Consider *low risk* of bias if the outcome variable(s) (e.g., behavioral, emotional, or cognitive outcome) is/are clearly stated and the rationale for its inclusion is reported.
- Consider *moderate risk* of bias if outcome definition is not (clearly) stated and/or rationale is not reported.
- Consider *high risk* of bias if the defined and described outcome variable(s) represent only a subset of a larger outcome set that are not reported in the current study.

5.2 Valid and Reliable Measurement of Outcome - The method of outcome measurement used is adequately valid and reliable to limit misclassification bias.

- Consider *low risk* of bias if outcome assessors are blind to participant group status.
- Consider *low* risk of bias if clinically-observed outcomes are used.
- Consider *low* risk of bias if validated patient-reported outcome measures are used.
- Consider *moderate risk* of bias if the validity and reliability of outcome measures have not been established.
- Consider *moderate risk* of bias if the outcome is measured based on clinical opinion of a medical examiner, with or without established diagnostic criteria.
- Consider *high risk* of bias if only self-reported outcomes are included (without the use of validated patient-reported outcome measures).
- Consider *high risk* of bias if an informant, such as a spouse, reports the outcome of interest.
- For neuroimaging studies, consider *high risk* of bias if only specific brain regions are examined and reported and no study protocol, preceding data collection, is available that specifies the *a priori* brain regions that were analyzed.

5.3 Method and Setting of Outcome Measurement - The method and setting of outcome measurement is the same for all study participants.

- Consider *low risk* of bias if all study participants experience the same method and setting of outcome measurement.
- Consider *moderate risk* of bias if no information, or little information, is reported about whether the method and setting of outcome measurement is the same for all participants.
- Consider *high risk* of bias if discrepancy exists in the method and setting of outcome measurement between groups with different levels of the prognostic variable (i.e., different age of first exposure).
- Consider *high risk* of bias if outcome assessors are not blind to participant group status.

5.4 Outcome Measurement Summary - Outcome of interest is adequately measured in study participants to sufficiently limit potential bias.

6 Study Confounding: To judge the risk of bias due to confounding (i.e., the effect of the prognostic factor is distorted by another factor that is related to the prognostic factor and the outcome).

6.1 Important Confounders Measured - All important confounders are measured:

- Consider *low risk* of bias if five of the following seven confounding variables are measured and reported: (1) prior concussion history, (2) diagnosed learning disability/ADHD/learning accommodations, (3) history of headaches or migraine/treatment for headaches or migraines, (4) mental health problems, (5) socioeconomic status or race/ethnicity, (6) duration of play, (7) education.
- Consider *moderate risk* of bias if no information, or a lack of information, about potentially relevant confounding variables is reported.
- Consider *high risk* of bias if fewer than five of the following five confounding variables are measured and reported: (1) prior concussion history, (2) diagnosed learning disability/ADHD/learning accommodations, (3) history of headaches or migraine/treatment for headaches or migraines, (4) mental health problems, (5) socioeconomic status or race/ethnicity, (6) duration of play, (7) education.

6.2 Definition of the confounding factor - Clear definitions of the important confounders measured are provided.

- Consider *low risk* of bias if confounding variables (e.g., learning disability) are clearly described including clear definitions that detail the diagnoses, level or severity, duration of experience, and/or time since experience (where applicable).
- Consider *moderate risk* of bias if confounding factors are not defined or poorly defined with little information provided.
- Consider *high risk* of bias if the definition provided does not reflect confounding variables measured.

6.3 Valid and Reliable Measurement of Confounders - Measurement of important confounders is adequately valid and reliable.

- Consider *low risk* of bias if measurement of the confounding variable is valid and reliable using objective methods (including validated, diagnostic criteria or health records).
- Consider *moderate risk* of bias if confounding variables are measured using retrospective self-report by the participant.

- Consider *high risk* of bias if an informant, such as a spouse, subjective reports confounders, instead of the participant himself/herself.

6.4 Method and Setting of Confounding Measurement - The method and setting of confounding measurement are the same for all study participants.

- Consider *low risk* of bias if identical method and setting of confounding measurement is used for all participants (i.e., for those with different levels of the prognostic factor – e.g., AFE < 12 years & AFE ≥ 12 years).
- Consider *moderate risk* of bias if method and setting of confounding factors are not defined or poorly defined.
- Consider *high risk* of bias if there are discrepancies in the method and setting of confounding measurement.

6.5 Method used for missing data - Appropriate methods are used if imputation is used for missing confounder data.

- Consider *low risk* of bias if the authors report no missing data for confounding variables.
- Consider *low risk* of bias if missing data for confounding variable(s) are reported and addressed by excluding the participant with missing confounding variable data.
- Consider *moderate risk* of bias if no information about missing data for confounding variables is reported.
- Consider *high risk* of bias if missing data for confounding variable(s) are reported and addressed using adjustment methods (e.g., missing data imputation), irrespective of method used.

6.6 Appropriate Accounting for Confounding - Important potential confounders are accounted for in the analysis.

- Consider *low risk* of bias if studies account for baseline confounding using stratification, regression, standardization, and inverse probability weighting. Each method depends on the assumption that there is no unmeasured or residual confounding.
- Consider *low risk* of bias if studies are designed in a manner to account for potentially confounding variables (e.g., matching for key variables, variable/group stratification, initial assembly of comparable group from study commencement).
- Consider *moderate or high risk* of bias if studies do not try to account for potentially confounding variables in the analyses (e.g., matching for key variables, variable/group stratification, initial assembly of comparable group from study commencement).

6.7 Study Confounding Summary - Important potential confounders are appropriately accounted for, limiting potential bias with respect to the relationship between the prognostic factor (AFE) and outcome.

7 Statistical Analysis & Reporting: To judge the risk of bias related to the statistical analysis and presentation of results.

7.1 Presentation of analytical strategy - There is sufficient presentation of data to assess the adequacy of the analysis.

- Consider *low risk* of bias if analytic strategy and data are sufficiently presented with or without an available pre-specified statistical analysis plan presented to assess the adequacy of the analysis.
- Consider *moderate risk* of bias if the statistical analysis appears appropriate (even in the absence of a pre-registered and available study protocol with statistical analysis plan) but there is suspicion that multiple analyses of the prognostic factor and outcome (or many outcomes) have been trialed with only one analytical approach reported.
- Consider *high risk* of bias if there is clear evidence (e.g., through assessment of a pre-registered study protocol or statistical analysis plan) that the prognostic factor and outcome were analyzed in multiple ways, but only one analytical approach, or a subset of analytical approaches, is fully reported (without justification), and the fully reported statistical analysis is likely to have been selected based on the results.

7.2 Model development strategy - The strategy for model building (i.e., inclusion of variables in the statistical model) is appropriate and is based on a conceptual framework or model.

- Consider *low risk* of bias if the analytic strategy is appropriate and clearly reports the independent, confounding, and dependent variables included in each statistical model.
- Consider *moderate risk* of bias if no information, or little information, is reported about the analytical strategy and the presence and sequence of independent, confounding, and dependent variables.
- Consider *moderate risk* of bias if the authors perform many comparisons between groups (e.g., using numerous outcome measures) and do not report adjusting for multiple comparisons that are performed.
- Consider *high risk* of bias if the analytic strategy is not appropriate or if one or more statistical test assumptions are clearly violated.

7.3 Reporting of results - There is no selective reporting of results.

- Consider *low risk* of bias if the reported results seem appropriate, complete, and are unlikely to have been selectively reported due to their nature, direction, and/or magnitude.
- Consider *low risk* of bias if both adjusted and unadjusted results are provided.
- Consider *moderate risk* of bias if there are moderate to high levels of suspicion that multiple outcome variables were included for analysis but selectively reported (i.e., selective outcome reporting/non-reporting).
- Consider *high risk* of bias if there is clear evidence (e.g., through reference of an available, pre-registered study protocol) that multiple outcome variables were included and selectively reported based on the observed result.

7.4 Statistical Analysis and Presentation Summary - The statistical analysis is appropriate for the design of the study, limiting potential for presentation of invalid or spurious results.

8 Overall Risk of Bias Judgment Criteria

- If all risk of bias domains are at ‘low’ risk of bias, consider an overall judgment of ‘low’ risk of bias.
- If there is at least one domain at ‘moderate’ risk of bias, consider an overall judgment of ‘moderate’ risk of bias.
- If there is at least one domain at ‘high’ risk of bias or multiple domains at ‘moderate’ risk of bias, consider an overall judgment of ‘high’ risk of bias.

References

1. Stamm JM, Bourlas AP, Baugh CM, et al. Age of first exposure to football and later-life cognitive impairment in former NFL players. *Neurology* 2015;84:1114-20.
2. Stamm JM, Koerte IK, Muehlmann M, et al. Age at First Exposure to Football Is Associated with Altered Corpus Callosum White Matter Microstructure in Former Professional Football Players. *J Neurotrauma* 2015;32:1768-76.
3. Schultz V, Stern RA, Tripodis Y, et al. Age at First Exposure to Repetitive Head Impacts Is Associated with Smaller Thalamic Volumes in Former Professional American Football Players. *J Neurotrauma* 2018;35:278-285.
4. Kaufmann D, Sollmann N, Kaufmann E, et al. Age at First Exposure to Tackle Football is Associated with Cortical Thickness in Former Professional American Football Players. *Cereb Cortex* 2021.
5. Alosco ML, Kasimis AB, Stamm JM, et al. Age of first exposure to American football and long-term neuropsychiatric and cognitive outcomes. *Transl Psychiatry* 2017;7:e1236.
6. Montenigro PH, Alosco ML, Martin BM, et al. Cumulative Head Impact Exposure Predicts Later-Life Depression, Apathy, Executive Dysfunction, and Cognitive Impairment in Former High School and College Football Players. *J Neurotrauma* 2017;34:328-340.
7. Alosco ML, Mez J, Tripodis Y, et al. Age of first exposure to tackle football and chronic traumatic encephalopathy. *Ann Neurol* 2018;83:886-901.
8. Solomon GS, Kuhn AW, Zuckerman SL, et al. Participation in Pre-High School Football and Neurological, Neuroradiological, and Neuropsychological Findings in Later Life: A Study of 45 Retired National Football League Players. *Am J Sports Med* 2016;44:1106-15.
9. Roberts AL, Pascual-Leone A, Speizer FE, et al. Exposure to American Football and Neuropsychiatric Health in Former National Football League Players: Findings From the Football Players Health Study. *Am J Sports Med* 2019;47:2871-2880.
10. Iverson GL, Terry DP, Caccese JB, et al. Age of First Exposure to Football Is Not Associated with Midlife Brain Health Problems. *J Neurotrauma* 2021;38:538-545.
11. Iverson GL, Caccese JB, Merz Z, et al. Age of first exposure to football is not associated with later-in-life cognitive or mental health problems. *Frontiers in Neurology, section Neurotrauma* in press.
12. Bryant BR, Narapareddy BR, Bray MJC, et al. The effect of age of first exposure to competitive fighting on cognitive and other neuropsychiatric symptoms and brain volume. *Int Rev Psychiatry* 2020;32:89-95.
13. Hunzinger KJ, Caccese JB, Costantini KM, et al. Age of First Exposure to Collision Sports Does Not Affect Patient Reported Outcomes in Women and Men Community Rugby Players. *Med Sci Sports Exerc* 2021.
14. Houck ZM, Asken BM, Bauer RM, et al. Academic aptitude mediates the relationship between socioeconomic status and race in predicting ImPACT scores in college athletes. *Clin Neuropsychol* 2020;34:561-579.
15. Brett BL, Huber DL, Wild A, et al. Age of First Exposure to American Football and Behavioral, Cognitive, Psychological, and Physical Outcomes in High School and Collegiate Football Players. *Sports Health* 2019;11:332-342.
16. Caccese JB, DeWolf RM, Kaminski TW, et al. Estimated Age of First Exposure to American Football and Neurocognitive Performance Amongst NCAA Male Student-Athletes: A Cohort Study. *Sports Med* 2019;49:477-487.

17. Caccese JB, Iverson GL, Cameron KL, et al. Estimated Age of First Exposure to Contact Sports Is Not Associated with Greater Symptoms or Worse Cognitive Functioning in Male U.S. Service Academy Athletes. *J Neurotrauma* 2020;37:334-339.
18. Caccese JB, Bodt BA, Iverson GL, et al. Estimated Age of First Exposure to Contact Sports and Neurocognitive, Psychological, and Physical Outcomes in Healthy NCAA Collegiate Athletes: A Cohort Study. *Sports Med* 2020;50:1377-1392.
19. Caccese JB, Houck Z, Kaminski TW, et al. Estimated age of first exposure to American football and outcome from concussion. *Neurology* 2020;95:e2935-e2944.
20. Caccese JB, Santos FV, Yamaguchi F, et al. Age of First Exposure to Soccer Heading and Sensory Reweighting for Upright Stance. *Int J Sports Med* 2020;41:616-627.
21. Asken BM, Houck ZM, Clugston JR, et al. Word-reading ability as a "hold test" in cognitively normal young adults with history of concussion and repetitive head impact exposure: A CARE Consortium Study. *Clin Neuropsychol* 2020;34:919-936.
